# Expanding the genetic toolkit: adenine and cytosine base editors for gene disruption in *Aspergillus niger*

**DOI:** 10.1186/s12934-026-02979-y

**Published:** 2026-03-26

**Authors:** Guoliang Yuan, Shuang Deng, Ziyu Dai, Beth A. Hofstad, Kyle R. Pomraning

**Affiliations:** 1 Chemical and Biological Processes Development Group, Pacific Northwest National Laboratory, Richland, Washington, 99354 USA; 2 Department of Energy Agile BioFoundry, Emeryville, CA USA

**Keywords:** CRIPSR, Base editing, Premature stop codon, Intron retention, Start codon mutation, *Aspergillus*

## Abstract

Despite revolutionizing fungal genetic engineering, conventional CRISPR/Cas9-mediated knockouts rely on DNA double-strand breaks (DSBs), which can cause unwanted insertions and deletions, chromosomal abnormalities, and cytotoxicity. Base editors such as adenine base editors (ABEs), which convert A‧T to G‧C, and cytosine base editors (CBEs), which convert C‧G to T‧A, offer a safer alternative by enabling predictable, target-specific single-nucleotide changes without introducing DSBs. To overcome the limitations of traditional genome editing in filamentous fungi, we developed efficient base-editing systems in *Aspergillus niger*. For the first time, we constructed an ABE in *A. niger*, achieving up to 80% editing efficiency and inducing predictable A-to-G mutations at the intended intron sites, disrupting gene function through mRNA mis-splicing. We also developed a highly efficient CBE system, capable of introducing premature stop codons with 50–100% efficiency. To broaden the editing scope, we implemented a Cas9-NG variant recognizing a relaxed PAM sequence requiring only a single guanine (G), enabling editing at start codons and splice sites. Leveraging this expanded scope, we established gene disruption approaches by targeting start codons via ABE-mediated A-to-G conversions (ATG-to-GTG and ATG-to-ACG) and CBE-mediated C-to-T conversion (ATG-to-ATA). Additionally, our base-editing systems enable multiplex gRNA delivery and marker-free editing of multiple genes. Collectively, the scope-expanding strategies increase the number of genes targetable for disruption by base-editing in *A. niger* by 26.3% and enable near-complete coverage of 96% of the coding genes. Overall, this work demonstrates the potential of ABE and CBE systems as versatile, efficient, and safer alternatives to DSBs-based gene disruption in filamentous fungi.

**Supplementary Information:**

The online version contains supplementary material available at 10.1186/s12934-026-02979-y.

## Introduction

Filamentous fungi play pivotal roles in biotechnology, agriculture, and medicine due to their remarkable capacity to produce enzymes, organic acids, and a wide range of bioactive secondary metabolites [3, 19]. The advent of CRISPR/Cas9 technology has significantly accelerated genetic engineering in these organisms, enabling targeted genome modifications and advancing the exploration of fungal biology and metabolic engineering. Among the earliest demonstrations of this system in filamentous fungi was the establishment of a plasmid-based CRISPR/Cas9 platform in *Aspergillus* species, which helped pave the way for broader application in industrially relevant fungi [17]. However, the conventional CRISPR/Cas9 approach relies on the introduction of DNA double-strand breaks (DSBs), which can result in undesired genomic instability, cellular toxicity, and off-target effects, particularly in organisms with less efficient DNA repair pathways such as filamentous fungi [7, 15]. These drawbacks highlight the need for alternative strategies that enable predictable nucleotide conversions without introducing DSBs.

Base editing, a powerful CRISPR-derived technique, circumvents the need for DSBs by enabling direct and irreversible conversion of single DNA bases. This technology employs catalytically impaired Cas proteins fused with DNA deaminases to achieve highly specific nucleotide substitutions. Among the most widely used are adenine base editors (ABEs), which convert A‧T base pairs to G‧C, and cytosine base editors (CBEs), which convert C‧G to T‧A. Notably, base editing activity is typically confined to a defined nucleotide window within the target protospacer, producing canonical edits at expected positions, as well as heterogeneous or bystander edits within the same window. While base editors have been broadly applied in mammalian cells, plants, and yeasts, their development and use in filamentous fungi have lagged behind significantly [6, 10, 23, 25]. To date, only CBEs have been reported in filamentous fungi, including *Aspergillus niger*, *Aspergillus nidulans*, and *Myceliophthora thermophila*, but ABEs have not yet been developed or demonstrated in these organisms [8, 13, 24, 31].

Several major challenges currently limit the broader adoption of base editing in filamentous fungi. First, multiplexing capabilities, such as the use of multiple guide RNAs (gRNAs) for simultaneous editing of multiple genes, remain poorly developed. Second, the targeting scope is constrained by the strict NGG protospacer-adjacent motif (PAM) requirement of SpCas9, and PAM-relaxed variants such as Cas9-NG or SpRY have not yet been implemented in filamentous fungal base editing systems. These limitations significantly narrow the targeting scope and hinder functional genomics and strain engineering applications.

**Fig. 1:**
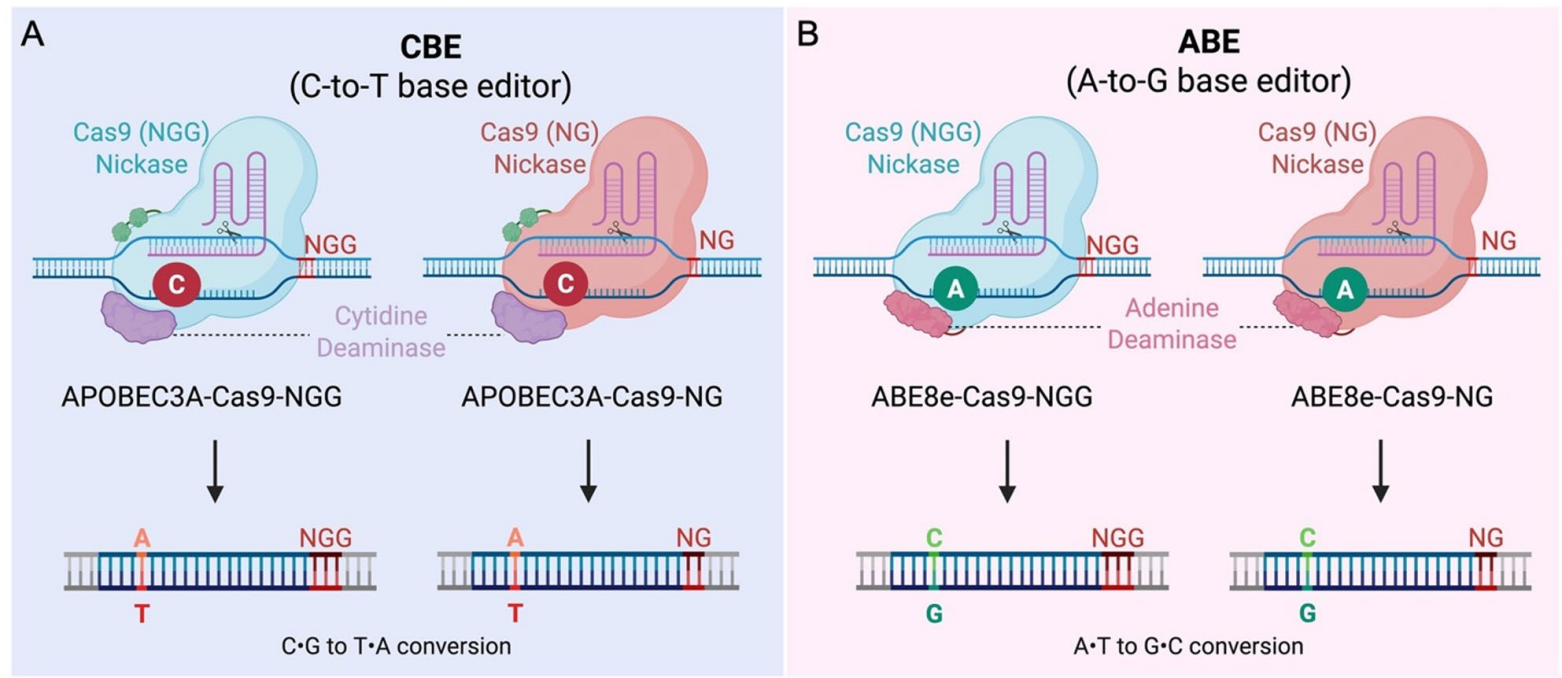
Illustration of current base editing platforms. (**A**). Cytosine base editors (CBEs) enable C-to-T editing (or G-to-A on the opposite strand) with NGG or NG PAMs. (**B**). Adenine base editors (ABEs) perform A-to-G conversion (or T-to-C on the opposite strand) using NGG or NG PAMs.

To address these gaps, we established a comprehensive base editing platform in *A. niger* that includes both ABE and CBE tools, supports multiplex gRNA expression for multi-locus editing, and incorporates PAM-relaxed Cas9 variants to expand the editable genome space (Fig. 1). By developing and optimizing these components, we seek to enable predictable, target-specific, and DSB-free genome editing in filamentous fungi and to advance their utility in both fundamental research and industrial biotechnology.

## Materials and methods

### Strains and culture conditions

For standard molecular cloning, *Escherichia coli* DH5α was employed as the host strain. The wild-type *A*. *niger* strain ATCC 11414 (originating from ATCC 1015/CBS 113.46/NRRL 328) was sourced from the American Type Culture Collection (Rockville, MD, USA). This strain was maintained and propagated on complete medium (CM) agar plates at 30 °C. To prepare spores, cultures were grown on CM for 4 days at 30 °C, then rinsed with sterile 0.4% Tween 80 solution to collect spores. The recipes for both complete and minimal media (MM) followed the formulation established by Bennett and Lasure [2]. CM contained (per liter): glucose, 20 g; trypticase peptone, 2 g; yeast extract, 5 g; casamino acids, 1 g; KH₂PO₄, 1.52 g; MgSO₄·7 H₂O, 0.52 g; KCl, 0.52 g; NaNO_3_, 6 g; supplemented with trace elements solution (22 mg ZnSO_4_·7 H_2_O; 11 mg H_3_BO_3_; 5 mg MnCl_2_·4 H_2_O; 5 mg FeSO_4_·7 H_2_O; 1.7 mg CoCl_2_·6 H_2_O; 1.6 mg CuSO_4_·5 H_2_O; 1.5 mg Na_2_MoO_4_·2 H_2_O; 50 mg Na_2_EDTA, pH6.5) and vitamin solution (1 mg each of biotin, pyridoxin-HCl, thiamine-HCl, riboflavin, p-aminobenzoic acid, and nicotinic acid ). MM contained (per liter): glucose, 10 g; NaNO₃, 6 g; KH₂PO₄, 1.52 g; MgSO₄·7 H₂O, 0.52 g; KCl, 0.52 g; supplemented with trace elements and vitamin solutions (The same as shown in CM). For solid media, agar was added at 15 g/L, and pH was adjusted to 6.5 for both CM and MM prior to autoclaving.

### Vector construction

A total of 36 vectors were constructed and used in this study (Table 1). The pGY18 vector (Addgene plasmid #221708) served as the empty backbone for cloning [28]. To construct pGY40, the AMA1-based backbone plasmid pGY18, which allows autonomous replication in filamentous fungi, was first linearized using the restriction enzymes BsaI and BamHI. The linearized vector was then assembled with two gBlocks Gene Fragments (51_gblocks and 52_gblocks) and three PCR products (PCR162185, PCR67166, and PCR165186) using HiFi DNA assembly. The gBlocks and PCR products were designed to provide specific functional elements for constructing the CBE vector. 51_gblocks contains the CBE coding sequence, and 52_gblocks provides the SV40 nuclear localization signal (NLS) fused to the C-terminus of nCas9. PCR162185 supplies the *tef1* promoter for driving nCas9 expression, PCR67166 contains the nCas9 coding sequence, and PCR165186 provides the *tef1* terminator for proper transcriptional termination of nCas9. Similarly, pGY49 was constructed by assembling the same linearized pGY18 with two different gBlocks Gene Fragments (74_gblocks and 75_gblocks) and the same set of PCR products. The pGY150 vector was generated using the same linearized pGY18, assembled with PCR products PCR426185, PCR427186, and the gBlock Gene Fragment 137_gblocks via HiFi DNA assembly. The pGY151 vector was generated using the same method. The remaining vectors were constructed by assembling the empty vector with the corresponding gBlocks or annealed oligonucleotides using Golden Gate assembly. All cloning PCR reactions were performed using Q5 Master Mix, following the manufacturer’s instructions. Cloning and restriction enzymes used in this study were obtained from New England Biolabs (NEB). All plasmid constructs were verified by Sanger sequencing. The gBlocks Gene Fragments were synthesized by IDT (see Supplementary Table S1). The experimental vectors pGY40 (Addgene plasmid #249718), pGY49 (Addgene plasmid #249719), pGY150 (Addgene plasmid #249722), and pGY151 (Addgene plasmid #249723) will be made available through Addgene. All gRNAs were designed through the CHOPCHOP online platform [11].

**Table 1:** All vectors used in this study

Vector name	Protospacer sequence	Purpose
pGY18	N/A	Empty vector of Cas9 system
pGY40	N/A	Empty vector of CBE-NGG system
pGY41	TS1: CGCTGACCAGCATGTTGACT TS2: ACGACGACTATGCTGGGACA	CBE-NGG base editing of *albA* gene
pGY49	N/A	Empty vector of ABE-NGG system
pGY50	TS1: CAGACCAGCGACATCGAAGC TS2: GGGAAGAGCTTCCGATGAGA	ABE-NGG base editing of *albA* gene
pGY60	TS1: ATATACGGTTTCGAAGAAGG TS2: TTCGAAACCGTATATCAGCG	ABE-NGG base editing of *albA* gene
pGY61	TS1: TCTGTCAGCATCCCACAATG TS2: AATTCATCAAGTACCGTAGG	ABE-NGG base editing of *albA* gene
pGY71	TCTGTCAGCATCCCACAATG	ABE-NGG base editing of *albA* gene
pGY72	AATTCATCAAGTACCGTAGG	ABE-NGG base editing of *albA* gene
pGY84	GTCAGCATCCCACAATGCGG	ABE-NGG base editing of *albA* gene
pGY85	TGTCAGCATCCCACAATGCG	ABE-NGG base editing of *albA* gene
pGY86	ACCAGCCTTCAGCTTTTACG	ABE-NGG base editing of *albA* gene
pGY87	ACGCAGTCGTTGGGCAAGAG	ABE-NGG base editing of *albA* gene
pGY88	AATCTTATGCAACTGAGCGC	ABE-NGG base editing of *albA* gene
pGY89	GAATCTTATGCAACTGAGCG	ABE-NGG base editing of *albA* gene
pGY90	TGTGGGATGCTGACAGATGC	ABE-NGG base editing of *albA* gene
pGY91	CGAATCTTAACGCAGTCGTT	ABE-NGG base editing of *albA* gene
pGY92	AACTTTAAACCCCCGCATTG	ABE-NGG base editing of *albA* gene
pGY94	AGATTCAAGGTGCTAATCAT	ABE-NGG base editing of *albA* gene
pGY95	TCAGACTTGTCAAGTATAGA	ABE-NGG base editing of *albA* gene
pGY96	CTGTCAGCATCCCACAATGC	ABE-NGG base editing of *albA* gene
pGY97	ACAATGCGGGGGTTTAAAGT	ABE-NGG base editing of *albA* gene
pGY98	GCTCAGTTGCATAAGATTCA	ABE-NGG base editing of *albA* gene
pGY99	ACTTTAAACCCCCGCATTGT	ABE-NGG base editing of *albA* gene
pGY100	CGTTAAGATTCGTACTAATC	ABE-NGG base editing of *albA* gene
pGY101	TTCAGCTTTTACGGGGATCT	ABE-NGG base editing of *albA* gene
pGY102	GACCAGCCTTCAGCTTTTAC	ABE-NGG base editing of *albA* gene
pGY103	TGACCAGCCTTCAGCTTTTA	ABE-NGG base editing of *albA* gene
pGY150	N/A	Empty vector of ABE-NG system
pGY151	N/A	Empty vector of CBE-NG system
pGY159	CCCTCCATGTTTGCGGAAGA	CBE-NG base editing of *albA* gene
pGY163	GCACTGCGACTGGGAATCTG	CBE-NG base editing of *albA* gene
pGY164	CCCTCCATGTTTGCGGAAGA	ABE-NG base editing of *albA* gene
pGY165	CAAACATGGAGGGTCCATCT	ABE-NG base editing of *albA* gene
pGY166	AACATGGAGGGTCCATCTCG	ABE-NG base editing of *albA* gene
pGY167	TGGTCAGACTTGTCAAGTAT	ABE-NG base editing of *albA* gene

TS1 and TS2 indicate target sites 1 and 2, respectively

### Protoplast transformation

The protoplast preparation method was mainly modified from a previous report [5]. Briefly, 30 mg/mL of VinoTaste Pro (Novonesis, Bagsværd, Denmark) was dissolved in the protoplast buffer (pH 5.5) containing 0.6 M (NH_4_)_2_SO_4_ and 50 mM maleic acid. The protoplasts were released at 30 °C and 80 rpm in a shaker-incubator for 2 to 3 h. The protoplasts were filtered through one layer of sterile Mirocloth (MilliporeSigma, Burlington, MA, USA) and concentrated by centrifugation at 4 °C and 800 × g for 10 min. The protoplasts were resuspended and washed in STC buffer (1 M sorbitol, 50 mM CaCl_2_, and 50 mM Tris-HCl, pH 8.0) twice. Finally, about 1 × 10^7^ protoplasts/mL were resuspended in STC buffer with 8% PEG 4000 (polyethylene glycol 4000). A polyethylene glycol (PEG)-based protocol was applied to introduce DNA into *A. niger* protoplasts. Approximately 100 µL of protoplasts was mixed with 1–2 µg of plasmid DNA (≤ 10 µL) in a sterile 15 mL centrifuge tube by gentle tapping and incubated on ice for 15 min. Then, 1 mL of 40% PEG was added, mixed gently, and kept at room temperature for another 15 min. Further, 5 mL of MM with 1 M sorbitol was added into the tubes, laid flat and shaken gently (~ 80 rpm) at 30 °C for 1 to 1.5 h. Finally, the transformed protoplasts were precipitated by centrifugation at 800 × g and 4 °C for 5 min. The supernatants were discarded, and the protoplast pellets were resuspended in 15 mL of MM containing 1 M sorbitol, 0.8% agar and 300 µg/mL geneticin (G418) at 50 °C, then poured onto a petri dish and incubated at 30 °C until new mycelium regeneration from protoplasts. For each transformation, two controls were included: (i) a negative control, in which protoplasts were mixed with autoclaved dH₂O and plated on selective medium, and (ii) a regeneration control (once per batch of protoplast preparation), in which a series of diluted protoplasts without the transformation were directly plated on non-selective medium to confirm optimal regeneration efficiency and rule out biases unrelated to transformation efficiency. In addition, during the initial validation of the base editors, strains expressing the editor plasmid without a targeting gRNA (pGY40, pGY49, pGY150, and pGY151) were included as negative controls.

### Single colony isolation

CRISPR editing efficiency was calculated as the fraction of isolated transformants carrying detectable on-target edits. To accurately measure this efficiency, individual colonies were randomly isolated directly from the transformation plates at an early time point, as described previously [28]. In the later stages of growth, the plates develop into a confluent mycelial lawn, making it impossible to distinguish individual colonies. Therefore, single-colony picking was performed 19–24 h after transformation, when colonies were still extremely small and not visible to the naked eye. At this stage, individual transformants could be reliably identified and isolated under a stereomicroscope. Using a sterile syringe needle, individual colonies were gently lifted from the transformation plate and transferred to slants containing 1.5 mL of MM supplemented with 0.8% agar and 200 µg/mL G418. The slants were incubated at 30 °C until sporulation occurred. The resulting spores were then used for genotyping and downstream analyses. This approach ensured that each isolate originated from an independent transformation event rather than from clonal expansion within a confluent lawn.

### Single spore isolation

Single spore isolation was performed to obtain genetically homogeneous strains. Conidia from each isolated colony were suspended in sterile 0.4% Tween 80. A1-µL aliquot of the spore suspension was diluted with 50 µL of sterile dH₂O and spread onto fresh CM plates, which were then incubated at 30 °C overnight until individual spores began to germinate. Under a dissecting microscope, a germinated spore, which was well separated, was picked and transferred to a new CM slant or plate using a sterile needle. The resulting colonies derived from a single spore were used for subsequent phenotypic and genotypic analyses.

### PCR genotyping

Spores were collected by washing with sterile 0.4% Tween 80 solutions. The harvested spores were then used directly for Squash-PCR genotyping, following established protocols described by Yuan et al. [27, 29]. Genotyping PCR was performed using GoTaq Green Master Mix (Promega, Madison, WI, USA). Each 20 µL reaction contained 1 µL of squashed spore solution as the DNA template. The PCR program started with an initial denaturation at 95 °C for 2 min, followed by 35 cycles of 95 °C for 30 s (denaturation), 55 °C for 30 s (annealing), and 72 °C for 1 min (extension). The reaction ended with a final extension at 72 °C for 5 min. All DNA oligos used for genotyping are listed in Supplementary Table S2. To validate base edits, PCR products were analyzed by Sanger sequencing. Chromatograms were manually examined and only reads with Phred scores ≥ 30 at the edited positions were considered. Ambiguous reads were re-sequenced. For each target site, wild-type genomic DNA was sequenced in parallel as a control to exclude PCR- or sequencing-induced artifacts.

### RNA extraction

Total RNA was extracted from *A*. *niger* spores as the biomass source. Spores were harvested and ground to a fine powder using a mortar and pestle that were precooled with liquid nitrogen. RNA extraction was then performed using the Maxwell^®^ RSC Plant RNA Kit (Promega, Madison, WI, USA) following the manufacturer’s instructions. During the automated extraction, DNase I was added into the given wells of a purification cartridge to remove residual genomic DNA, ensuring that subsequent analyses reflect mRNA rather than contaminating DNA. This procedure ensured isolation of high-quality RNA suitable for downstream applications.

### cDNA synthesis

First-strand complementary DNA (cDNA) was synthesized from purified total RNA using the SuperScript™ III First-Strand Synthesis System (Thermo Fisher Scientific, Waltham, MA, USA), following the manufacturer’s instructions. Briefly, up to 1 µg of total RNA was mixed with oligo(dT)₍₂₀₎, denatured at 65 °C for 5 min, and then chilled on ice. Reverse transcription was carried out at 50 °C for 50 min in the presence of SuperScript™ III reverse transcriptase and RNaseOUT™ inhibitor. The reaction was terminated by heating at 85 °C for 5 min. The resulting first-strand cDNA was stored at − 20 °C until further use.

### Sanger sequencing

PCR products were purified with the QIAquick Gel Extraction Kit (QIAGEN, Germantown, MA, USA) and then sent to GENEWIZ (South Plainfield, NJ, USA) for sequencing. The same primers used for genotyping PCR were also used for Sanger sequencing.

## Results

### Development and validation of CBE-mediated base editing using multiplexed RNAs

**Fig. 2:**
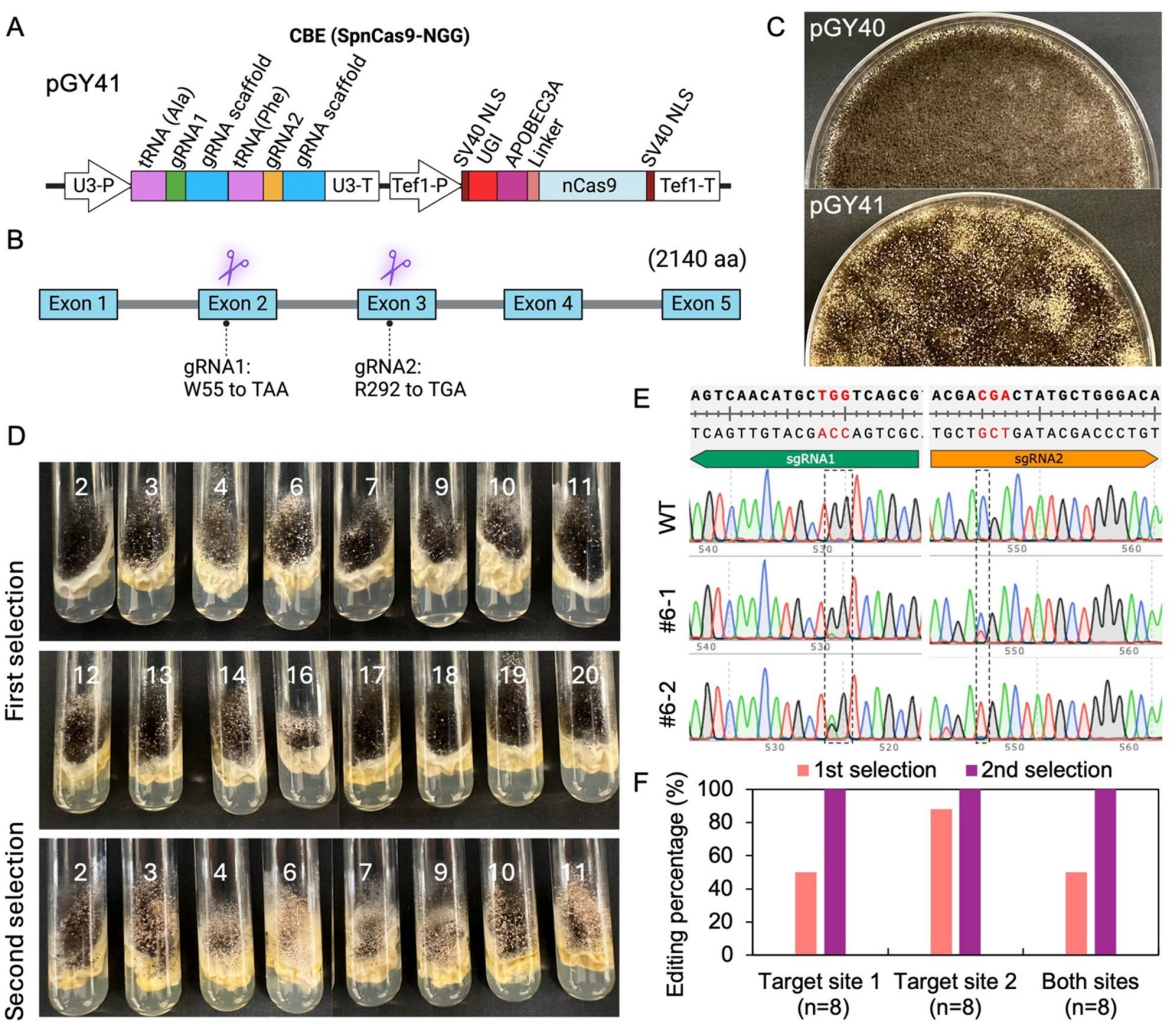
Development CBE base editor function with NGG PAMs in *A. niger*. (**A**). Schematic of the CBE base editor vector design. (**B**). Target sites within the *albA* gene for vector pGY41. (**C**). Phenotypes of *albA* base-edited colonies harboring pGY41 on the primary transformation plate, with pGY40 as the control, six days post-transformation. (**D**). Phenotype of edited colonies after single colony isolation. Individual colonies were isolated 19–24 h after transformation (see Sect. 2.4). Two rounds of selection were performed sequentially to enrich cells stably modified at both positions. (**E**). C-to-T (or G-to-A on the opposite strand) edits confirmed by Sanger sequencing. #6−1 is colony 6 from the first round of selection, and #6−2 is the same colony 6 that underwent the second round of selection. Dashed boxes highlight the nucleotide positions targeted for editing. A non-canonical on-target edit outside the canonical window is also observed. The codon targeted for editing is indicated in red. (**F**). Analysis of editing efficiency (%) for CBE-mediated base editing.

To establish efficient cytosine base editing in *A*. *niger*, we constructed a CBE vector designed to target multiple loci within the genome simultaneously. The base editor vector incorporates the cytidine deaminase APOBEC3A fused to a catalytically impaired nickase Cas9 (nCas9) capable of recognizing NGG PAM sequences (Fig. 2A). This system enables predictable C-to-T (or G-to-A on the opposite strand) conversions without introducing DSBs. We selected the *albA* gene as a reporter target due to its easily observable phenotypic change from pigmented to white spores upon mutation [4]. Using vector pGY41, two target sites within *albA* were simultaneously edited through multiplexed gRNAs: gRNA1 converted the TGG corresponding to the 55th amino acid tryptophan (W) to a TAG nonsense mutation in exon 2, and gRNA2 for the CGA of 292nd amino acid arginine (R) to TGA nonsense mutation in exon 3 (Fig. 2A and B). The multiplexed gRNAs were expressed using a tRNA-processing system, which has previously been demonstrated to support efficient multiplexed gRNA expression in *A. niger* [12, 28].

Six days after protoplast transformation with vector pGY41, the primary transformation plates displayed numerous white colonies with distinct pigmentation changes, consistent with *albA* disruption and indicative of successful CBE-mediated editing events (Fig. 2C). These visible phenotypes provided a convenient and reliable marker for identifying edited clones. However, at this late stage, the dense colony growth makes it impossible to distinguish or isolate individual colonies. Therefore, single colony isolation was performed much earlier, at 19–24 h after protoplast transformation, when colonies remained well separated. Consistent with the phenotypes observed on the primary plates, white spores were also present in all the single isolated colonies grown on slants (Fig. 2D). To verify the molecular nature of the edits, Sanger sequencing was performed on single isolated colonies using DNA prepared by Squash-PCR [27, 29]. The results confirmed expected C-to-T conversions at both target cytosines, generating nonsense codons TGG (W55) to TAG, corresponding to a G-to-A change on the complementary strand, and CGA (R292) to TGA (Fig. 2E and Supplementary Fig. S1). These findings demonstrated the specificity and efficiency of the multiplexed CBE approach in editing multiple sites simultaneously. Although 50% of the single isolated colonies showed the expected edits at both target sites, the low edited peak signals relative to wild-type peaks in the Sanger sequencing profiles indicate a very low intra-colony editing frequency (i.e., only a small subpopulation of nuclei carried the edit) (Supplementary Fig. S1). Consequently, obtaining pure mutants through single spore isolation would be challenging.

To further enrich for cells harboring edits at both loci, a second round of selection was performed by inoculating spores from the initially isolated single colonies onto fresh selection medium, without additional transformation. This strategy improved the completeness of base editing at the target sites. For instance, colony #6−1 from the first round showed partial editing at both sites, while its derivative, #6−2, obtained after the second selection round, exhibited complete editing at site 2 and substantial improvement at site 1 (Fig. 2E), demonstrating the benefit of sequential selection in recovering fully edited clones. A single non-canonical on-target edit outside the canonical window was also observed in #6−2 (Fig. 2E). After the second round of selection, every isolated single colony exhibited the expected edits at both target sites (Fig. 2F and Supplementary Fig. S1). These results indicate that dual-target editing can be achieved at the colony level, while individual colonies may still contain a mixture of canonical and non-canonical edits within the target protospacer. Collectively, these results establish RNA-guided CBE as a useful tool for functional multiplexed base editing in *A. niger*.

### Establishment and characterization of ABE-mediated base editing

**Fig. 3:**
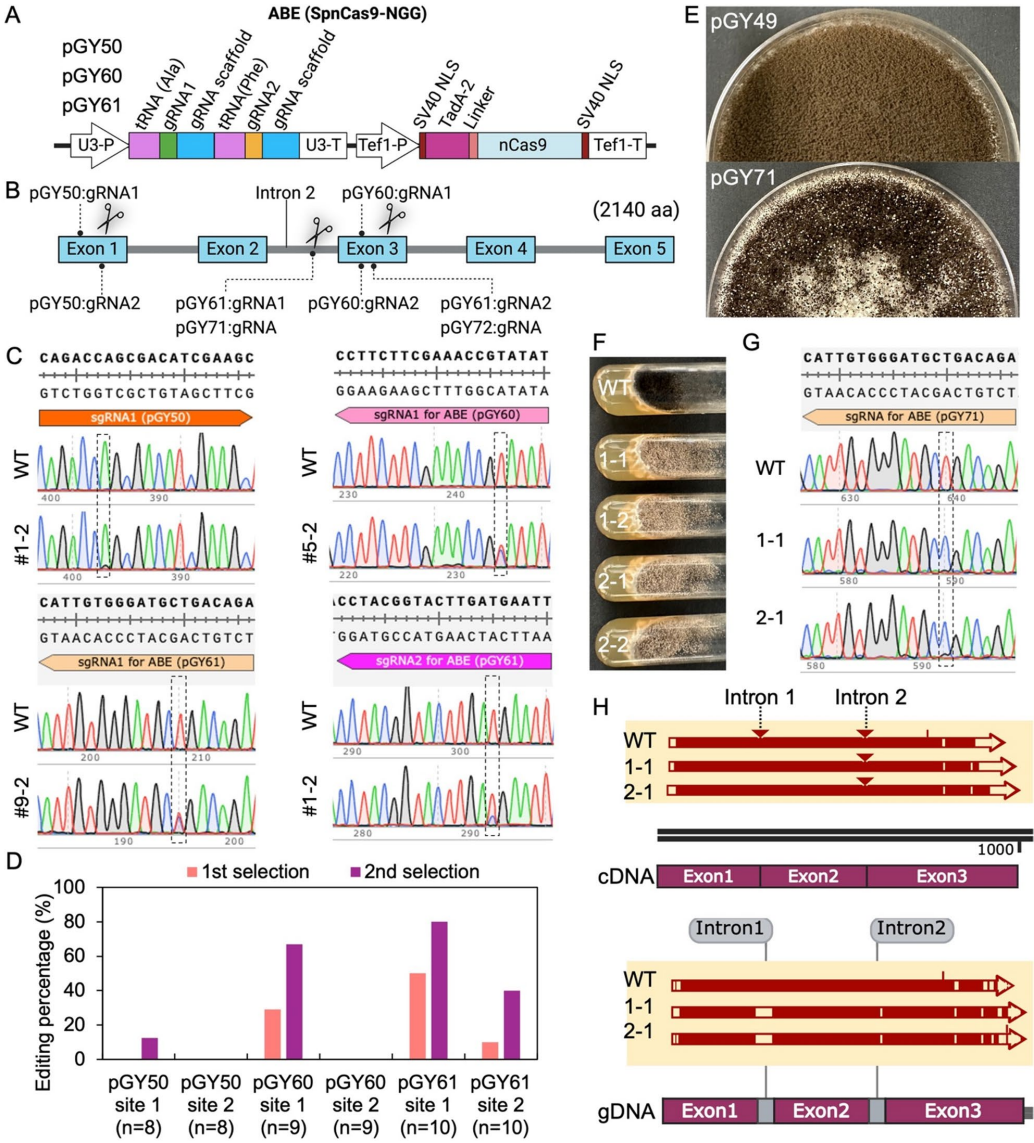
Development ABE base editor function with NGG PAMs in *A*. *niger*. (**A**). Schematic of the ABE base editor vector design. (**B**). Target sites within the *albA* gene for vectors pGY50, pGY60, pGY61, pGY71, and pGY72. (**C**). A-to-G conversion (or T-to-C on the opposite strand) confirmed by Sanger sequencing. All colonies are single isolated colonies from the primary one that were subjected to a second round of selection. Dashed boxes highlight the nucleotide positions targeted for editing. (**D**). Editing efficiency (%) analysis for ABE-mediated base editing, with two rounds of enrichment for edited events. (**E**). Phenotypes of *albA* base-edited colonies generated using pGY71, with pGY49 as the control, on the primary transformation plate. (**F**). Phenotype of edited colonies after single spore isolation of pGY71. 1–1 and 1–2 are sub-colonies of original colony 1; 2−1 and 2–2 are sub-colonies of original colony 2. (**G**). T-to-C conversion on the opposite strand confirmed by Sanger sequencing. Dashed boxes highlight the nucleotide positions targeted for editing. (**H**). Sequence alignment of cDNA from two selected colonies (1–1 and 2−1) with both the cDNA and genomic DNA (gDNA) of the wild-type strain, using WT gDNA as the control. In the alignment, a triangle (△) indicates a long insertion, a small white gap represents a point mutation or small deletion, a large white gap indicates a long deletion, and the arrow shows the alignment direction. The point mutation induced by ABE occurred in intron 2.

To expand the base editing toolkit for genetic engineering in filamentous fungi, we developed an adenine base editor (ABE) system capable of predictable A-to-G conversions. The ABE vector was constructed by fusing a nCas9 with an evolved adenine deaminase domain, TadA-2, and was optimized to recognize NGG PAM sequences for targeted editing (Fig. 3A). To evaluate the performance of the ABE system, multiple sites within the *albA* gene were selected, including adenines located in both exonic and intronic regions. A series of ABE vectors (pGY50, pGY60, pGY61, pGY71, and pGY72) were constructed, and each vector contained one or two gRNA expression cassettes targeting the indicated sites (Fig. 3B). Following protoplast transformation, isolated single colonies were screened by Squash-PCR, and Sanger sequencing was used to confirm A-to-G (or T-to-C on the complementary strand) conversions at the intended positions, validating the functionality of the ABE system in *A. niger*.

However, initial editing efficiencies varied across different loci, ranging from undetectable to approximately 50% (Fig. 3D and Supplementary Fig. S2), suggesting locus-dependent differences in accessibility or editing context. Given that sequential selection had previously enhanced editing outcomes in the CBE system, we applied the same strategy to ABE-edited strains. Specifically, spores from isolated primary colonies were reinoculated onto fresh selection medium to enrich for fully edited subpopulations. The second round of enrichment led to substantial increases in editing efficiency, with some loci reaching up to 80% as quantified in the post-selection colonies (Fig. 3C, D and Supplementary Fig. S2), demonstrating the benefit of iterative selection for improving ABE performance.

Interestingly, white spore colonies were observed on the primary transformation plate of pGY61 (Supplementary Fig. S3). Since pGY61 contains two gRNAs targeting both intron 2 and exon 3 of the *albA* gene, it was initially unclear whether the observed phenotype resulted from editing of the intron, the exon, or both. To examine the individual contributions of these two target sites, we constructed two additional vectors: pGY71, targeting only intron 2, and pGY72, targeting only exon 3 (Fig. 3B). After protoplast transformation, white colonies were observed on the transformation plate of pGY71 but not on the plate of pGY72, indicating that mutation within intron 2 was primarily responsible for the observed loss-of-function phenotype (Fig. 3E).

To further characterize the editing outcome, two white colonies (#1 and #2) from the pGY71 transformation plate were selected and subjected to single-spore isolation. This resulted in two subclones per colony: #1–1 and #1–2 from colony #1, and #2−1 and #2–2 from colony #2 (Fig. 3F). Squash-PCR and Sanger sequencing confirmed successful A-to-G editing at the targeted adenine, corresponding to a T-to-C conversion on the complementary strand (Fig. 3G). Furthermore, cDNA sequencing revealed intron 2 retention in both subclones #1–1 and #2−1, demonstrating that the ABE-induced mutation disrupted proper intron splicing (Fig. 3H).

Together, these results demonstrate that the ABE system enables predictable and effective A-to-G base editing in *A. niger*, with editing efficiencies enhanced through sequential selection. Importantly, we show that ABE can be used not only for direct gene modification but also for functional gene inactivation through targeted disruption of intron splicing. The successful induction of intron retention by a single base conversion highlights the potential of ABE-mediated editing to manipulate RNA processing and gene expression in filamentous fungi. These findings establish ABE as a versatile and powerful addition to the fungal genome engineering toolbox, complementing CBE and enabling new strategies for functional genomics and strain development.

### Gene inactivation via intron mis-splicing induced by ABE editing

**Fig. 4:**
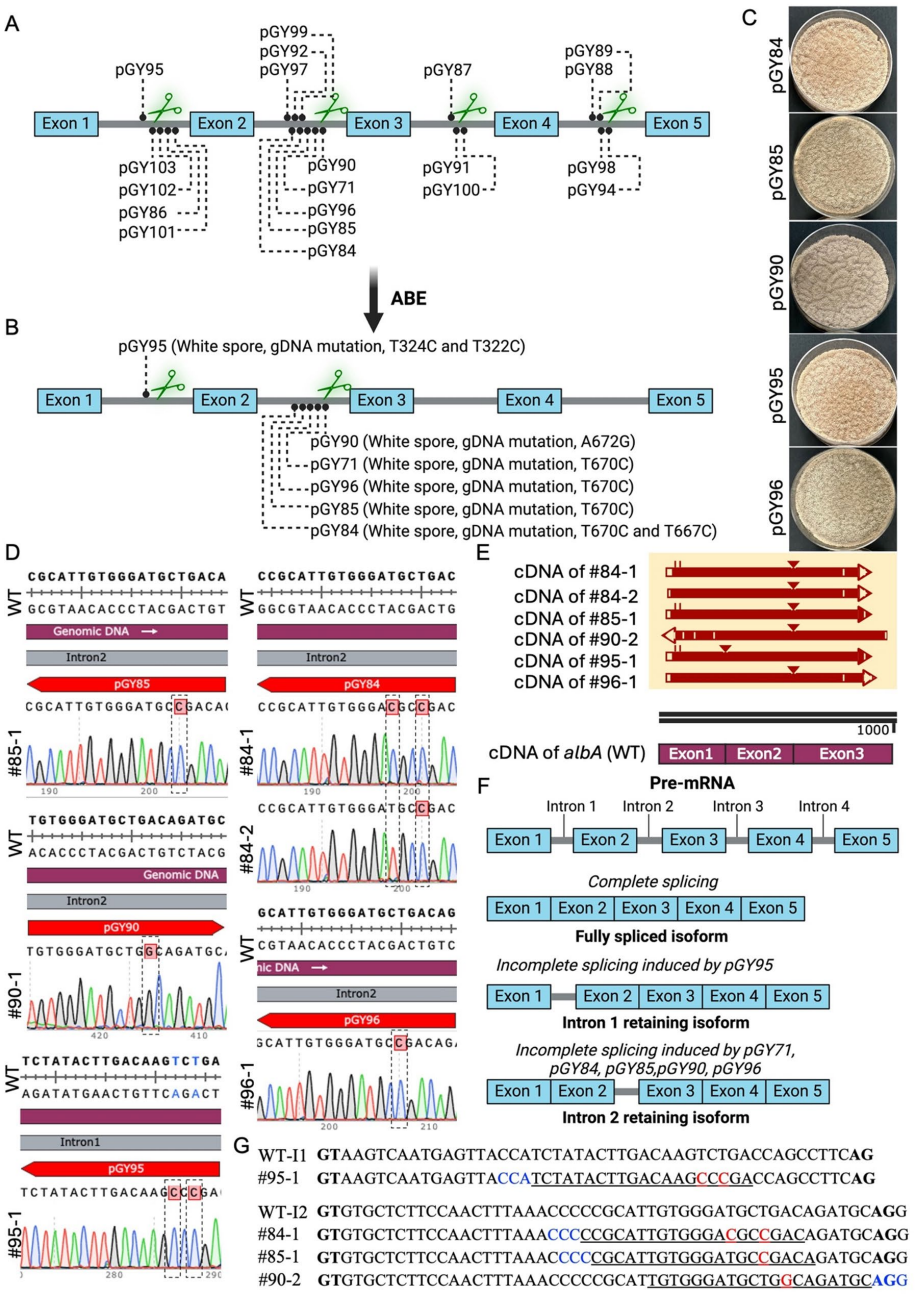
Gene inactivation induced by ABE-mediated intron mis-splicing. (**A**). Selected target sites within the intron of the *albA* gene. (**B**). Identification of target sites leading to gene inactivation via ABE editing. (**C**). Phenotype of edited colonies following single spore isolation of pGY84, pGY85, pGY90, pGY95, and pGY96 constructs. (**D**). A-to-G conversion (or T-to-C on the opposite strand) confirmed by Sanger sequencing. Dashed boxes highlight the nucleotide positions targeted for editing. (**E**). Sequence alignment of the cDNA from selected colonies compared with the cDNA of the wild-type strain. Within the alignment, long insertions are marked with a triangle (△), and the arrow reflects the sequence’s orientation. The triangle in colony #95−1 indicates intron 1 retention, whereas triangles in the remaining colonies indicate intron 2 retention. The ABE editor–derived mutation occurred in intron 1 in colony #95−1 and in intron 2 in the remaining colonies. (**F**). Intron retention resulting from ABE base editing. (**G**). Sequence alignment of the gDNA from selected colonies with the intron sequence of the wild-type strain. Intron 1 (I1) and Intron 2 (I2) are indicated. Canonical splice sites are shown in bold: GT at the 5′ end of the intron (splice donor) and AG at the 3′ end (splice acceptor). The gRNA is underlined, the PAM is highlighted in blue, and the nucleotide conversions are highlighted in red.

Building on the finding that ABE-induced base editing can disrupt intron splicing and cause intron retention, we investigated adenine base editing as a novel strategy for targeted gene inactivation via intron mis-splicing. Specifically, we leveraged ABE to target putative critical adenines within all introns of the *albA* gene in *A. niger*, with the goal of disrupting key splicing signals such as branch points, or regulatory elements essential for accurate intron removal (Fig. 4A). To achieve this, a total of 20 gRNAs were designed to target adenines across introns 1 through 4.

Of the 20 gRNAs tested, 6 resulted in functional gene inactivation via ABE-mediated editing at intronic sites, as determined by screening multiple target loci (Fig. 4B). In addition to pGY71 described above, transformed colonies with the constructs pGY84, pGY85, pGY90, pGY95, and pGY96 displayed distinct phenotypic changes, indicating the *albA* loss-of-function, both on the primary transformation plates and after single spore isolation (Fig. 4C and Supplementary Fig. S4).

Sanger sequencing validated efficient A-to-G (or T-to-C on the opposite strand) conversions at the targeted intronic adenines, confirming predictable base editing within the non-coding regions (Fig. 4D). To assess the impact of these edits on transcript processing, cDNA from selected edited colonies was aligned against wild-type cDNA sequences. The alignments revealed aberrant splicing patterns, notably intron retention events that are absent in the wild type (Fig. 4E and F). These retained introns are likely the result of disrupted splicing signals caused by the single-nucleotide substitutions introduced by ABE editing, highlighting the sensitivity of splice site recognition to even subtle sequence changes. Genomic DNA alignment further confirmed that these mutations resided within the targeted introns, specifically intron 1 (I1) and intron 2 (I2) (Fig. 4G). The retained intron typically introduced premature stop codons or frameshifts into the coding sequence, ultimately leading to loss-of-function alleles. These findings reinforce the idea that targeted single-base modifications within non-coding regions can effectively disrupt gene function without altering coding exons, offering a minimally invasive yet highly effective tool for functional gene studies.

Together, these results demonstrate that ABE can be strategically applied to induce gene inactivation by perturbing intron splicing mechanisms, providing a predictable and effective approach to functional genomics studies in filamentous fungi.

### Expansion of CBE and ABE base editing to NG PAMs

**Fig. 5:**
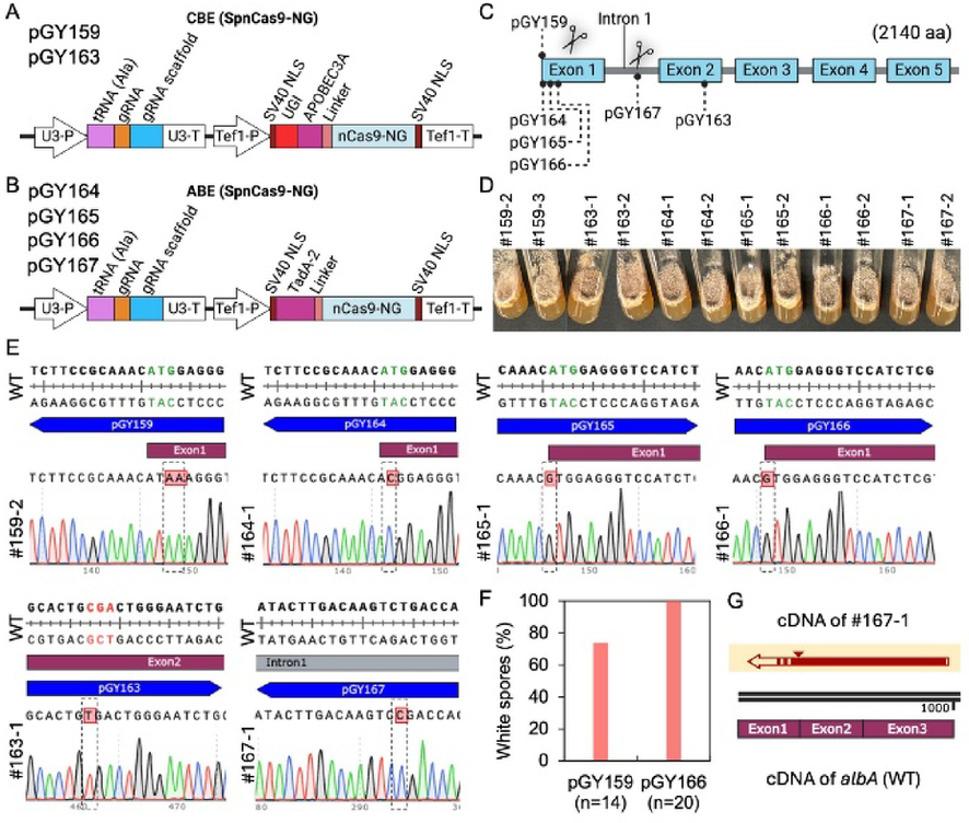
Development CBE and ABE base editor function with NG PAMs in *A. niger*. (**A**). Schematic of the CBE base editor vector design. (**B**). Schematic of the ABE base editor vector design. (**C**). Target sites within the *albA* gene for vectors pGY159, pGY163, pGY164, pGY165, pGY166, and pGY167. (**D**). Phenotype of edited colonies after single spore isolation. (**E**). C-G to T-A editing induced by CBE and A-T to G-C editing induced by ABE, confirmed by Sanger sequencing. pGY159 and pGY163 are CBEs. pGY164 to pGY167 are ABEs. Dashed boxes highlight the nucleotide positions targeted for editing. The codons targeted for editing are indicated in red and green. (**F**). White spores (%) analysis of CBE-NG-mediated base editing using pGY159 and ABE-NG-mediated base editing using pGY166. (**G**). Sequence alignment of the cDNA from colony #167-1 with the cDNA of the wild-type. The triangle indicates intron 1 retention.

To expand the targeting range of base editing in *A. niger*, we developed cytosine and adenine base editors that recognize NG PAM sequences beyond the canonical NGG PAM. This broadens the flexibility and versatility of genome editing. Both editors utilize a SpCas9-NG variant with the mutations R1335V, L1111R, D1135V, G1218R, E1219F, A1322R, and T1337R, which we refer to as VRVRFRR [16]. The CBE and ABE vector designs incorporating NG PAM compatibility are illustrated in Fig. 5A and B, respectively. Using these vectors, multiple target sites within the *albA* gene including the start codon, intron 1, and exon 2 were selected and targeted with vectors pGY159 and pGY163 for CBE NG, as well as pGY164 through pGY167 for ABE NG (Fig. 5C).

White spores, indicating successful *albA* editing, were observed on the primary transformation plates for all tested vectors (Supplementary Fig. S5A). After single spore isolation, these white-spore colonies consistently exhibited the expected *albA* mutant phenotype (Fig. 5D). Sanger sequencing confirmed the presence of the expected C‧G to T‧A conversions introduced by the CBE NG editors (pGY159 and pGY163), as well as A‧T to G‧C conversions mediated by the ABE NG editors (pGY164 to pGY167) (Fig. 5E). These results validate the high targeting precision of both base editing platforms at NG PAM sites.

Following a single selection round, a high proportion of colonies exhibited the expected white-spore phenotype associated with *albA* disruption, with pGY159 (CBE-NG) reaching 74% (*n* = 14) and pGY166 (ABE-NG) reaching 100% (*n* = 20), representing the highest phenotypic mutation frequencies among the constructs tested (Fig. 5F and Supplementary Fig. S5B). Other plasmids exhibited relatively low phenotypic mutation frequencies, as indicated by the low proportion of white spores on the primary transformation plate (Supplementary Fig. S5A). In this experiment, mutation frequency was assessed based on phenotypic observation rather than sequencing confirmation. To investigate the downstream effects of base editing at the transcript level, cDNA sequencing of colony #167-1 revealed the anticipated base changes, along with retention of intron 1. This intron retention is likely a consequence of disrupted splicing signals introduced by the ABE NG-mediated edit, further supporting the functional impact of the targeted modifications (Fig. 5G).

Taken together, these findings confirm the successful adaptation of cytosine and adenine base editors to recognize NG PAM sequences, thereby overcoming a key constraint associated with NGG-limited Cas9 editing. By broadening the editable sequence space, this advancement significantly enhances the flexibility and applicability of base editing in *A*. *niger*, enabling more versatile genetic manipulation for functional genomics and strain engineering.

## Discussion

**Fig. 6:**
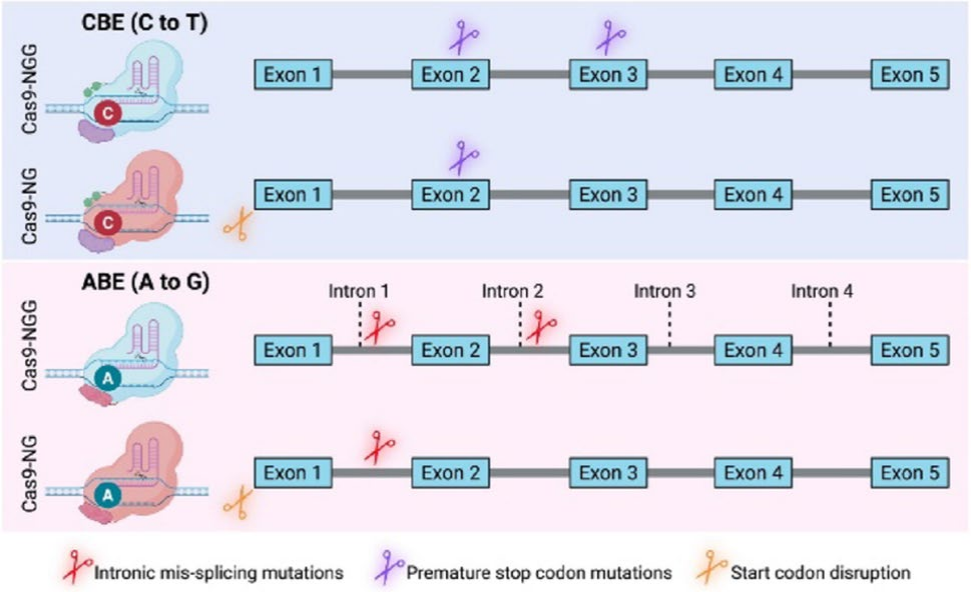
Illustration of gene inactivation induced by CBEs and ABEs with NGG or NG PAMs in *A. niger*. CBE (NGG or NG) was used to induce premature stop codon mutations, while ABE (NGG or NG) was employed to induce intronic mis-splicing mutations. Additionally, CBE (NG) and ABE (NG) were utilized to disrupt start codons.

In this study, we established a comprehensive base-editing platform for *A*. *niger* that includes both cytosine and adenine base editors, supports multiplexed gRNA delivery, and is compatible with relaxed PAM requirements through SpCas9-NG. We report, for the first time in filamentous fungi, the successful implementation of an ABE system, capable of introducing predictable A-to-G conversions with editing efficiencies reaching up to 80% [13]. In parallel, we optimized a CBE system that achieved high-efficiency editing (50–100%) at multiple genomic loci. Collectively, these tools enabled predictable and effective gene disruption through premature stop codons, intron mis-splicing, and start codon mutations without inducing DSBs (Fig. 6).

Notably, the base editing efficiencies achieved in this study are comparable to those reported for base editors in other filamentous fungi, where efficiencies have been shown to vary substantially across target loci and editor architectures. For example, cytosine base editing in *A. niger* has been reported to range from ~ 40% to near-complete editing depending on the genomic locus and selection strategy [8], while studies in other fungi likewise report broad variability in base-editing or CRISPR-mediated editing efficiencies across targets and systems [14, 30, 31].

Compared to conventional CRISPR/Cas9-mediated knockouts, base editors offer several compelling advantages. While DSB-based approaches rely on error-prone repair mechanisms and can result in unpredictable insertions or deletions, base editing introduces specific, single-nucleotide changes in a clean and controlled manner [13, 18, 22]. This precision significantly reduces the risk of chromosomal rearrangements or cytotoxicity and allows for subtle modifications that can alter gene function or regulation without disturbing surrounding sequences [20]. Importantly, our results demonstrate that base editing can be harnessed not only to disrupt coding sequences directly but also to modulate non-coding regulatory elements, such as introns, thereby expanding the functional genomics toolkit in the filamentous fungi. In practice, certain phenotypic outcomes highlight the differences between base editing and DSB-based strategies. Although some single colonies produced white spores after the first round of base editing-mediated selection, none reached the fully white spore phenotype observed in *albA* knockouts generated by CRISPR-Cas9 or Cas12a [28]. This underscores a key limitation of base editing: functional outcomes depend strongly on target site selection, editing window constraints, and the biological consequence of the nucleotide change, which can lead to mixed or partially edited nuclei. It is important to clarify that the stereo microscope was used only for early-stage single-colony isolation and not for phenotype-based screening. Edited mutants were confirmed by Sanger sequencing, and thus the workflow does not rely on visible phenotypic changes.

A key methodological insight from our work is the advantage of performing a second round of selection after transformation. In both ABE and CBE experiments, this enrichment step substantially increased both the frequency and completeness of editing events by maintaining the stability of AMA1-based autonomous replicating CRISPR plasmids and sustaining expression of the editing machinery [9, 28]. Similar iterative selection strategies have been reported previously in classical CRISPR/Cas9 workflows aimed at gene knockout. For example, in *Penicillium digitatum*, successive rounds of selection using an AMA1-based plasmid targeting the *pksP/arp2* locus markedly increased knockout efficiency [21]. In our study, we demonstrate that this approach is likewise highly effective for SpCas9-based ABE and CBE base editing in *A. niger*, as cells that escape editing during the initial selection can still undergo subsequent editing while retaining the plasmids inside the cells. Importantly, this iterative strategy offers a faster and more practical alternative to new transformations, as partially edited clones were readily converted into fully edited genotypes after a second selection, an advantage in fungal systems with variable transformation efficiency and editing outcomes.

A major limitation of base editing systems is their dependence on a strict PAM located at a precise position relative to the target site [26]. To address this constraint, an important contribution of this study is the successful adaptation of both ABE and CBE systems to function with Cas9-NG, a PAM-relaxed variant that recognizes a broader NG PAM sequence [16]. To our knowledge, this is the first demonstration of NG-PAM-compatible base editing in filamentous fungi. The broader targeting range afforded by the Cas9-NG variant dramatically increases the number of editable sites within the genome, making it feasible to target previously inaccessible loci such as start codons or specific splice sites. In the *A*. *niger* genome, relaxing the PAM specificity increases the number of genes targetable for disruption by introducing a premature stop codon within the 5’ quarter of the coding sequence by 25%, and by mutating the start codon by 216% (Table 2). Overall, 11,432 of the 11,910 predicted genes (96.0%) are targetable for disruption using the PAM-relaxed CBE and ABE systems. We confirmed the functionality and efficiency of this approach through targeted editing of introns and start codons using both CBE-NG and ABE-NG vectors. These experiments resulted in predictable single-base substitutions at intended sites, which are not accessible by regular ABE-NGG or CBE-NGG editors due to PAM constraints. While expanding PAM compatibility greatly increases the number of editable sites, it may also raise the likelihood of off-target editing, as NG-recognizing Cas9 variants can tolerate a broader range of genomic sequences. Although no obvious off-target consequences were observed in our phenotypic analyses or Sanger sequencing of the targeted loci, future genome-wide studies will be important to comprehensively assess unintended editing events associated with NG-PAM base editors in filamentous fungi.

Overall, our study highlights several key considerations and potential improvements for base editing in *A. niger*: (i) editing efficiencies remain highly locus- and gRNA-dependent, (ii) base editing cannot generate large deletions and may be less suitable for complete gene knockouts in loci with alternative splicing, and (iii) iterative selection or homokaryon purification may be required to achieve fully edited, homogeneous strains. Future work could focus on further improving the system by optimizing editor expression levels, systematically evaluating gRNA design rules specific to *A. niger*, narrowing or shifting the editing window, or coupling base editing to complementary DSB-based approaches for enhanced functional knockout.

In summary, our work establishes a versatile, predictable, and DSB-free genome editing platform for *A. niger* by leveraging both ABE and CBE technologies. These tools enable efficient gene inactivation through multiple strategies, including nonsense mutation, splice site disruption, and start codon modification, and support multiplexing and expanded PAM recognition. This platform significantly advances the genetic toolbox available for filamentous fungi and paves the way for more sophisticated functional genomics, metabolic engineering, and strain improvement strategies in these industrially and biologically important organisms.

**Table 2:** Predicted number of *A*. *niger* genes targetable for disruption by base editing

Editor	Start codon mutation		Premature stop codon introduction		Any disruption	
	NGG	NG	NGG	NG	NGG	NG
CBE	841	3033	8814	11,056	9054 (76.0%)	11,373 (95.5%)
ABE	954	3332	–	–	954 (8.0%)	3332 (28.0%)
Either	1133	3575	8814	11,056	9146 (76.8%)	11,432 (96.0%)

“Any disruption” represents the union of start codon mutations and premature stop codon introductions. Percentages are relative to the 11,910 annotated genes. NGG and NG represent the PAM sequences compatible with each base editor. Gene counts were predicted using the *Aspergillus niger* ATCC 1015 v4.0 genome assembly and annotation from JGI [1]

## Supplementary Information

Below is the link to the electronic supplementary material.

Supplementary Material 1: Fig. S1. C-to-T (or G-to-A on the opposite strand) edits were confirmed by Sanger sequencing. Fig. S2. A-to-G conversion (or T-to-C on the opposite strand) confirmed by Sanger sequencing. Fig. S3. Phenotypes of *albA* base-edited colonies generated with pGY61, observed under a stereo microscope on the primary transformation plate. Fig. S4. Phenotypes of *albA* base-edited colonies generated using pGY84, pGY85, pGY90, pGY95, and pGY96 on the primary transformation plates. Fig. S5. Phenotypes of *albA* base-edited colonies generated through CBE-NG and ABE-NG systems. Table S1. All gBlocks used in this study. Table S2. All DNA oligos used in this study.

## Data Availability

All data generated or analyzed in this study are included in the published article and its supplementary materials. Plasmid constructs will be made available through Addgene ( [https://www.addgene.org/](https:/www.addgene.org) ).

## References

[1] Andersen MR, Salazar MP, Schaap PJ, van de Vondervoort PJ, Culley D, Thykaer J, Frisvad JC, Nielsen KF, Albang R, Albermann K, et al. Comparative genomics of citric-acid-producing Aspergillus niger ATCC 1015 versus enzyme-producing CBS 513.88. Genome Res. 2011;21:885–97. 21543515 10.1101/gr.112169.110 PMC3106321

[2] Bennett J, Lasure L. 1991. Growth media. *More gene manipulations in fungi*: 441–447.

[3] Cairns TC, Zheng X, Zheng P, Sun J, Meyer V. Turning Inside Out: Filamentous Fungal Secretion and Its Applications in Biotechnology, Agriculture, and the Clinic. J Fungi. 2021;7:535. 10.3390/jof7070535 PMC8307877 34356914

[4] Chiang Y-M, Meyer KM, Praseuth M, Baker SE, Bruno KS, Wang CCC. Characterization of a polyketide synthase in Aspergillus niger whose product is a precursor for both dihydroxynaphthalene (DHN) melanin and naphtho-γ-pyrone. Fungal Genet Biol. 2011;48:430–7. 21176790 10.1016/j.fgb.2010.12.001 PMC3118676

[5] Debets A, Bos C. Isolation of small protoplasts from Aspergillus niger. Fungal Genet Rep. 1986;33:24.

[6] Gaudelli NM, Komor AC, Rees HA, Packer MS, Badran AH, Bryson DI, Liu DR. Programmable base editing of A•T to G•C in genomic DNA without DNA cleavage. Nature. 2017;551:464–71. 29160308 10.1038/nature24644 PMC5726555

[7] Guo C, Ma X, Gao F, Guo Y. Off-target effects in CRISPR/Cas9 gene editing. Front Bioeng Biotechnol. 2023;11:1143157. 36970624 10.3389/fbioe.2023.1143157 PMC10034092

[8] Huang L, Dong H, Zheng J, Wang B, Pan L. Highly efficient single base editing in Aspergillus niger with CRISPR/Cas9 cytidine deaminase fusion. Microbiol Res. 2019;223–225:44–50. 31178050 10.1016/j.micres.2019.03.007

[9] Katayama T, Nakamura H, Zhang Y, Pascal A, Fujii W, Maruyama J-i. Forced Recycling of an AMA1-Based Genome-Editing Plasmid Allows for Efficient Multiple Gene Deletion/Integration in the Industrial Filamentous Fungus < i>Aspergillus oryzae. Appl Environ Microbiol. 2019;85:e01896–01818. 30478227 10.1128/AEM.01896-18 PMC6344613

[10] Komor AC, Kim YB, Packer MS, Zuris JA, Liu DR. Programmable editing of a target base in genomic DNA without double-stranded DNA cleavage. Nature. 2016;533:420–4. 27096365 10.1038/nature17946 PMC4873371

[11] Labun K, Montague TG, Krause M, Torres Cleuren YN, Tjeldnes H, Valen E. CHOPCHOP v3: expanding the CRISPR web toolbox beyond genome editing. Nucleic Acids Res. 2019;47:W171–4. 31106371 10.1093/nar/gkz365 PMC6602426

[12] Li C, Zhou J, Rao S, Du G, Liu S. Visualized Multigene Editing System for Aspergillus niger. ACS Synth Biol. 2021;10:2607–16. 34555894 10.1021/acssynbio.1c00231

[13] Li X-H, Lu H-Z, Yao J-B, Zhang C, Shi T-Q, Huang H. Recent advances in the application of CRISPR/Cas-based gene editing technology in Filamentous Fungi. Biotechnol Adv. 2025;81:108561. 40086675 10.1016/j.biotechadv.2025.108561

[14] Ma B, Li Y, Wang T, Li D, Jia S. 2025. Advances in CRISPR/Cas9-Based Gene Editing in Filamentous Fungi. J Fungi (Basel) 11. 10.3390/jof11050350 PMC12112844 40422684

[15] Nishida K, Kondo A. CRISPR-derived genome editing technologies for metabolic engineering. Metab Eng. 2021;63:141–7. 33307189 10.1016/j.ymben.2020.12.002

[16] Nishimasu H, Shi X, Ishiguro S, Gao L, Hirano S, Okazaki S, Noda T, Abudayyeh OO, Gootenberg JS, Mori H, et al. Engineered CRISPR-Cas9 nuclease with expanded targeting space. Science. 2018;361:1259–62. 30166441 10.1126/science.aas9129 PMC6368452

[17] Nødvig CS, Nielsen JB, Kogle ME, Mortensen UH. A CRISPR-Cas9 System for Genetic Engineering of Filamentous Fungi. PLoS ONE. 2015;10:e0133085. 26177455 10.1371/journal.pone.0133085 PMC4503723

[18] Pacesa M, Pelea O, Jinek M. Past, present, and future of CRISPR genome editing technologies. Cell. 2024;187:1076–100. 38428389 10.1016/j.cell.2024.01.042

[19] Patil RH, Patil MP, Maheshwari VL. Chapter 5 - Bioactive Secondary Metabolites From Endophytic Fungi: A Review of Biotechnological Production and Their Potential Applications. Studies in Natural Products Chemistry. R Atta ur. Volume 49. Elsevier; 2016. pp. 189–205.

[20] Rees HA, Liu DR. Base editing: precision chemistry on the genome and transcriptome of living cells. Nat Rev Genet. 2018;19:770–88. 30323312 10.1038/s41576-018-0059-1 PMC6535181

[21] Ropero-Pérez C, Marcos JF, Manzanares P, Garrigues S. Increasing the efficiency of CRISPR/Cas9-mediated genome editing in the citrus postharvest pathogen Penicillium digitatum. Fungal Biol Biotechnol. 2024;11:8. 39003486 10.1186/s40694-024-00179-0 PMC11245846

[22] Shen Q, Ruan H, Zhang H, Wu T, Zhu K, Han W, Dong R, Ming T, Qi H, Zhang Y. Utilization of CRISPR-Cas genome editing technology in filamentous fungi: function and advancement potentiality. Front Microbiol. 2024;15:1375120. 38605715 10.3389/fmicb.2024.1375120 PMC11007153

[23] Tan J, Zhang F, Karcher D, Bock R. Engineering of high-precision base editors for site-specific single nucleotide replacement. Nat Commun. 2019;10:439. 30683865 10.1038/s41467-018-08034-8 PMC6347625

[24] Tian Y, Xu Q, Pang M, Ma Y, Zhang Z, Zhang D, Guo D, Wang L, Li Q, Li Y, et al. CRISPR-Cas9 Cytidine-Base-Editor Mediated Continuous In Vivo Evolution in Aspergillus nidulans. ACS Synth Biol. 2025;14:621–8. 39865728 10.1021/acssynbio.4c00716

[25] Yao T, Yuan G, Lu H, Liu Y, Zhang J, Tuskan GA, Muchero W, Chen J-G, Yang X. 2023. CRISPR/Cas9-based gene activation and base editing in Populus. Hortic Res 10. 10.1093/hr/uhad085 PMC10266945 37323227

[26] Yu SY, Birkenshaw A, Thomson T, Carlaw T, Zhang LH, Ross CJD. Increasing the Targeting Scope of CRISPR Base Editing System Beyond NGG. Crispr j. 2022;5:187–202. 35238621 10.1089/crispr.2021.0109

[27] Yuan G, Czajka JJ, Dai Z, Hu D, Pomraning KR, Hofstad BA, Kim J, Robles AL, Deng S, Magnuson JK. Rapid and robust squashed spore/colony PCR of industrially important fungi. Fungal Biology Biotechnol. 2023;10:15. 10.1186/s40694-023-00163-0 PMC10329332 37422681

[28] Yuan G, Deng S, Czajka JJ, Dai Z, Hofstad BA, Kim J, Pomraning KR. 2024. CRISPR-Cas9/Cas12a systems for efficient genome editing and large genomic fragment deletions in Aspergillus niger. Front Bioeng Biotechnol Volume 12–2024. 10.3389/fbioe.2024.1452496 PMC11521959 39479294

[29] Yuan G, Salalila A, Hwang S, Deng ZD, Deng S. 2025. An innovative high-throughput genome releaser for rapid and efficient PCR screening. Front Bioeng Biotechnol Volume 13–2025. 10.3389/fbioe.2025.1547909 PMC11975962 40200958

[30] Zhang C, Li N, Rao L, Li J, Liu Q, Tian C. Development of an Efficient C-to-T Base-Editing System and Its Application to Cellulase Transcription Factor Precise Engineering in Thermophilic Fungus < i>Myceliophthora thermophila. Microbiol Spectr. 2022;10:e02321–02321. 35608343 10.1128/spectrum.02321-21 PMC9241923

[31] Zhao F, Sun C, Liu Z, Cabrera A, Escobar M, Huang S, Yuan Q, Nie Q, Luo KL, Lin A, et al. Multiplex Base-Editing Enables Combinatorial Epigenetic Regulation for Genome Mining of Fungal Natural Products. J Am Chem Soc. 2023;145:413–21. 36542862 10.1021/jacs.2c10211 PMC10162584

