# Supplementary material for: Expanding the genetic toolkit: adenine and cytosine base editors for gene disruption in Aspergillus niger
Source: Microb Cell Fact. 2026 Mar 26;25:116. doi: 10.1186/s12934-026-02979-y (PMC13141642; doi:10.1186/s12934-026-02979-y)
Supplement: Supplementary file 1 — Supplementary Material 1: Fig. S1. C-to-T (or G-to-A on the opposite strand) edits were confirmed by Sanger sequencing. Fig. S2. A-to-G conversion (or T-to-C on the opposite strand) confirmed by Sanger sequencing. Fig. S3. Phenotypes of albA base-edited colonies generated with pGY61, observed under a stereo microscope on the primary transformation plate. Fig. S4. Phenotypes of albA base-edited colonies generated using pGY84, pGY85, pGY90, pGY95, and pGY96 on the primary transformation plates. Fig. S5. Phenotypes of albA base-edited colonies generated through CBE-NG and ABE-NG systems. Table S1. All gBlocks used in this study. Table S2. All DNA oligos used in this study. [file 12934_2026_2979_MOESM1_ESM.docx]

**Expanding the Genetic Toolkit: Adenine and Cytosine Base Editors for Gene Disruption in *Aspergillus Niger***

Guoliang Yuan^a,b,^*, Shuang Deng^a,b^, Ziyu Dai^a,b^, Beth A. Hofstad^a,b^ and Kyle R. Pomraning^a,b,^*

^a^Chemical and Biological Processes Development Group, Pacific Northwest National Laboratory, Richland, Washington 99352, United States

^b^US Department of Energy Agile BioFoundry, Emeryville, California, United States

*Corresponding authors: Guoliang Yuan (guoliang.yuan@pnnl.gov); Kyle R. Pomraning (kyle.pomraning@pnnl.gov)


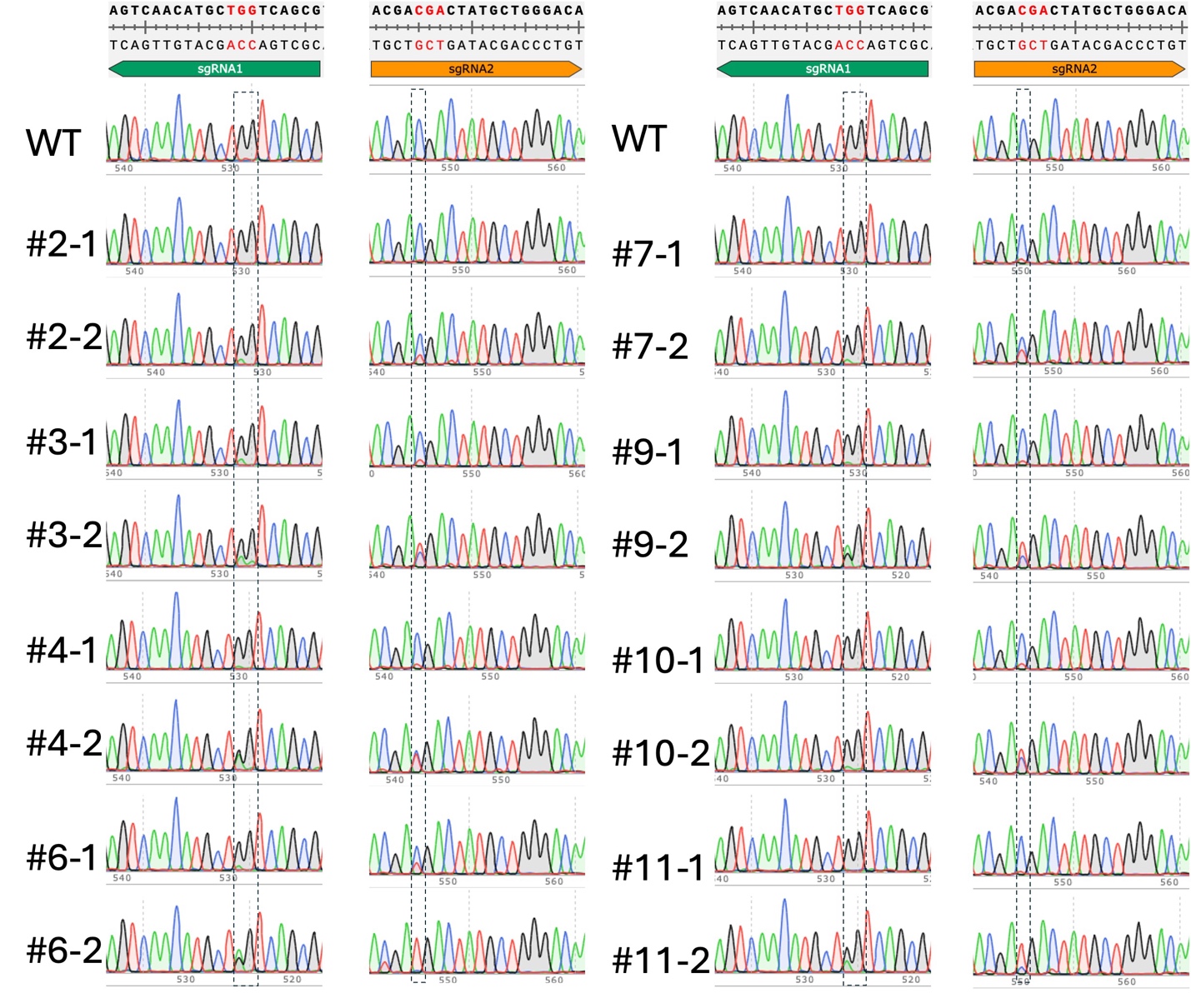


**Supplementary Fig. S1. C-to-T (or G-to-A on the opposite strand) edits were confirmed by Sanger sequencing.** Two rounds of selection were performed sequentially to enrich cells stably modified at both positions. -1 represents the first round of selection, and -2 represents the second round of selection. Dashed boxes highlight the nucleotide positions targeted for editing. The codon targeted for editing is indicated in red. Non-canonical on-target edits outside the canonical window are also observed in #6-2.

**
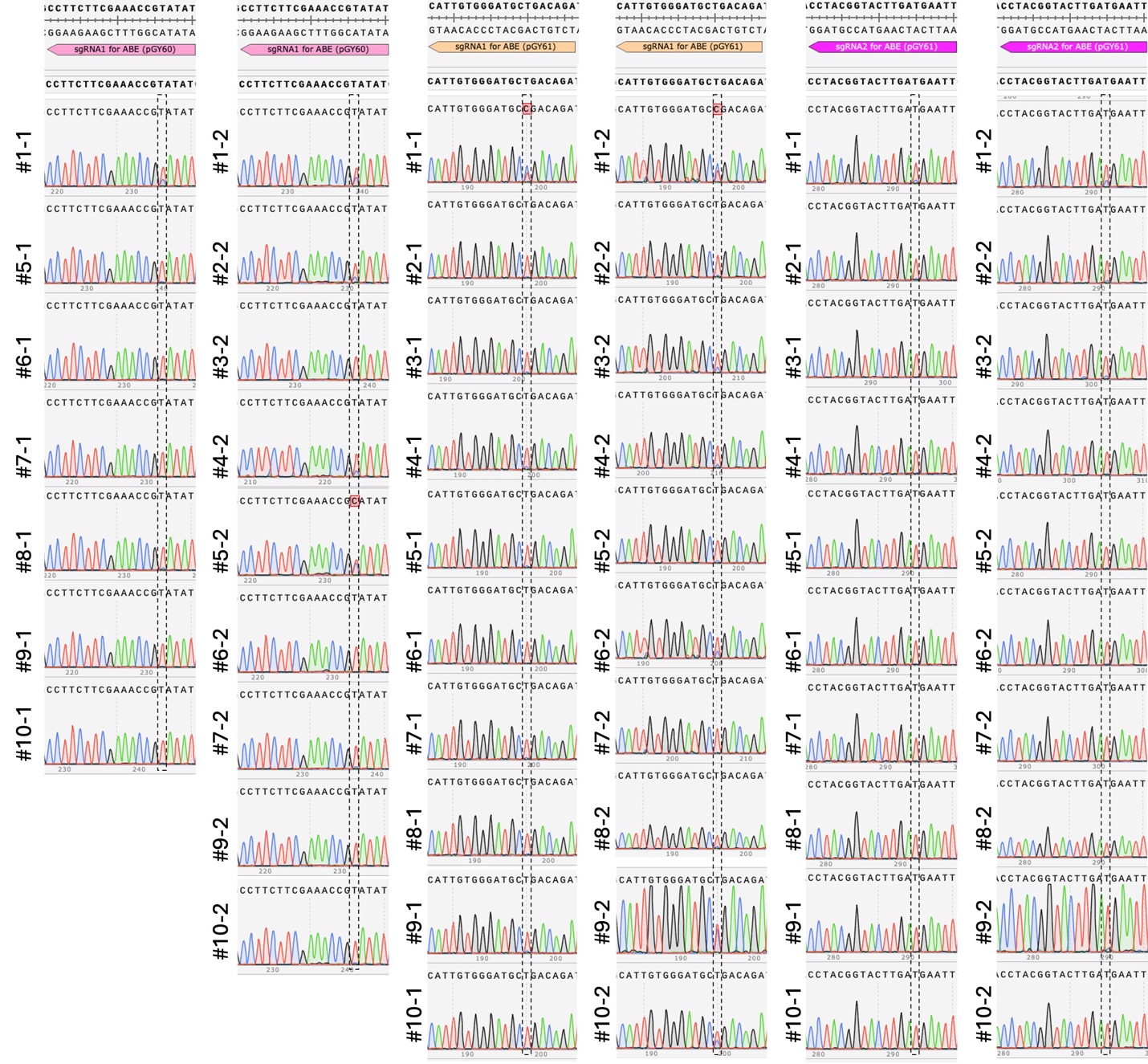
**

**Supplementary Fig. S2. A-to-G conversion (or T-to-C on the opposite strand) confirmed by Sanger sequencing.** Two rounds of selection were performed sequentially to enrich cells stably modified at both positions. -1 represents the first round of selection, and -2 represents the second round of selection. Dashed boxes highlight the nucleotide positions targeted for editing.


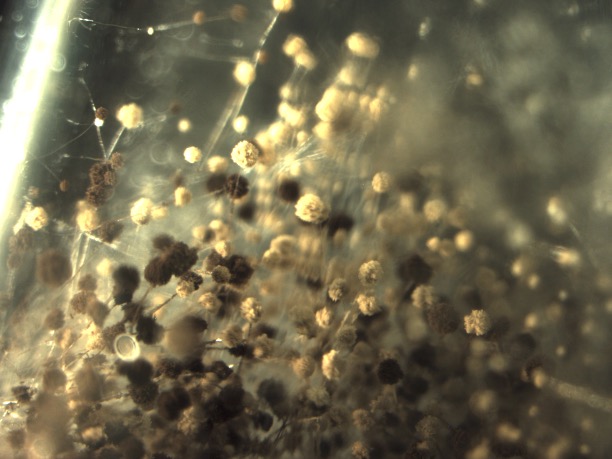


**Supplementary Fig. S3. Phenotypes of *albA* base-edited colonies generated with pGY61, observed under a stereo microscope on the primary transformation plate.**


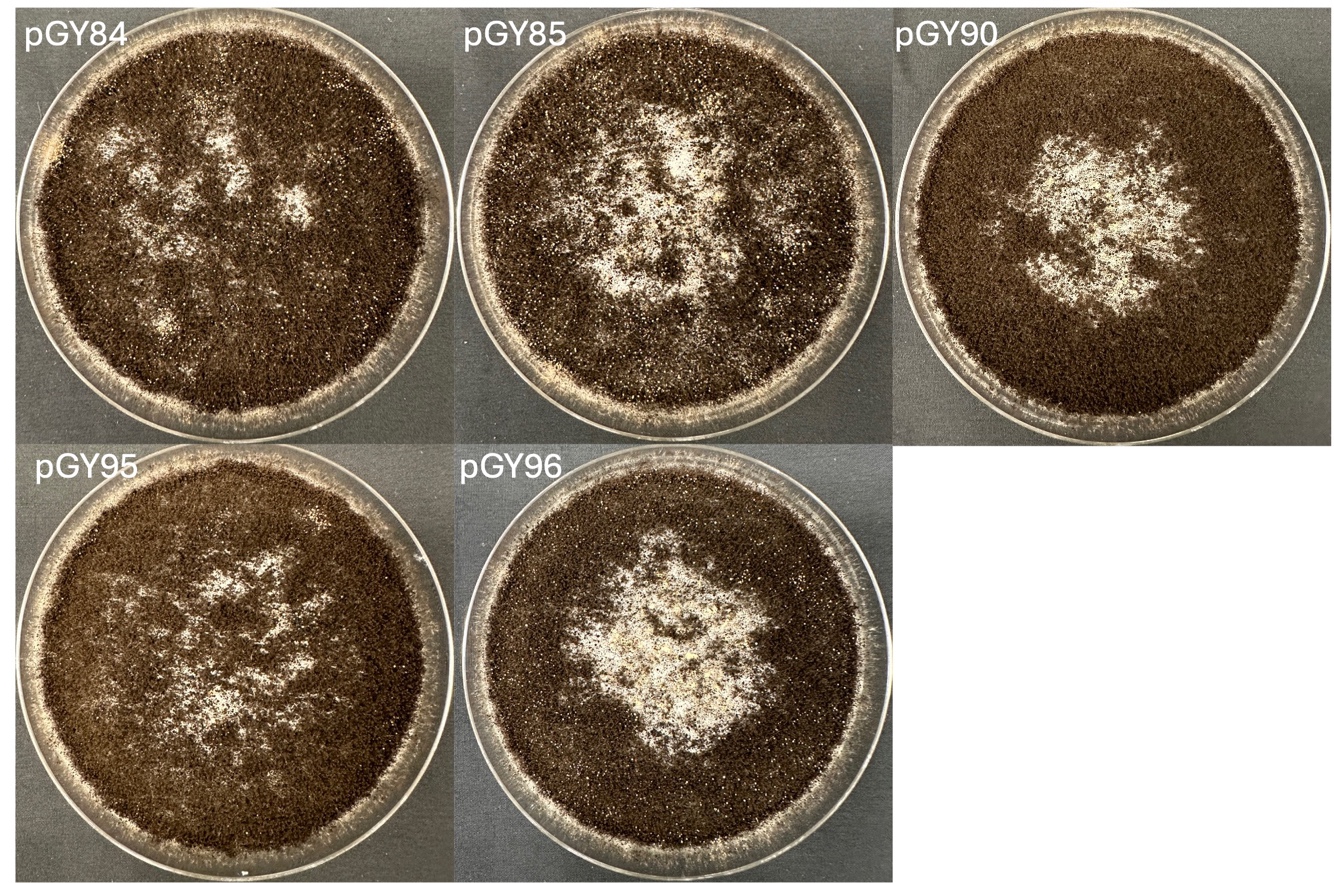


**Supplementary Fig. S4. Phenotypes of *albA* base-edited colonies generated using pGY84, pGY85, pGY90, pGY95, and pGY96 on the primary transformation plates.**

**
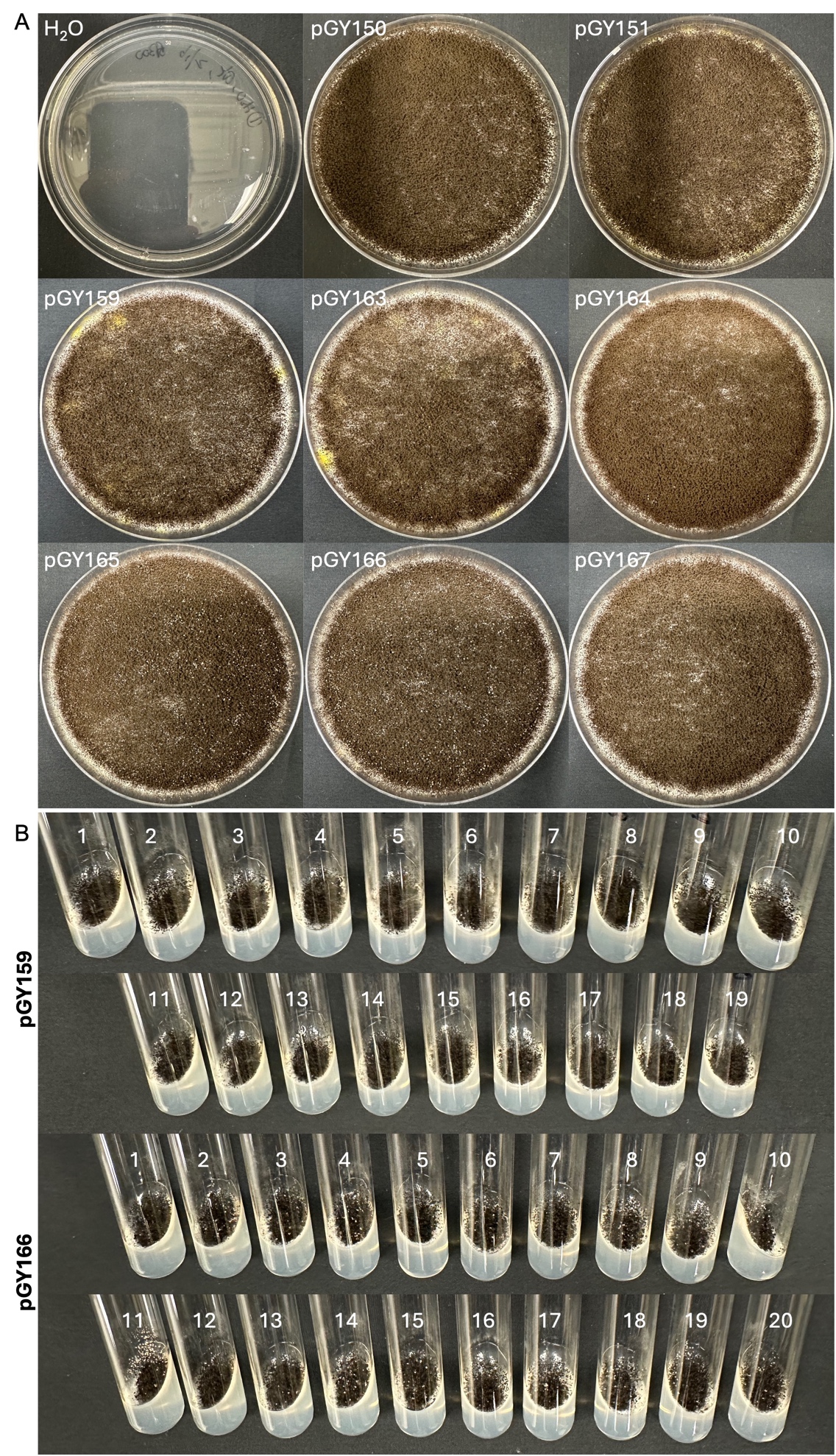
**

**Supplementary Fig. S5. Phenotypes of *albA* base-edited colonies generated through CBE-NG and ABE-NG systems. (A)** Phenotypes of *albA* base-edited colonies generated on the primary transformation plates with H_2_O, pGY150 and pGY151 as the controls. **(B)** Phenotypes of edited colonies after single colony isolation.**Supplementary** **Table S1. All gBlocks used in this study.**

| **Name** | **Sequence** | **Purpose** |
| --- | --- | --- |
| 51_gBlocks | AGTATCTGAGCACTTCTCCCTTTTATATTCCACAAAACATAACACGCGGCCGCATGGGACCTAAGAAAAAGAGGAAGGTGGCGGCCGCTACTAATCTGAGCGACATCATTGAGAAGGAGACTGGGAAACAGCTGGTCATTCAGGAGTCCATCCTGATGCTGCCTGAGGAGGTGGAGGAAGTGATCGGCAACAAGCCAGAGTCTGACATCCTGGTGCACACCGCCTACGACGAGTCCACAGATGAGAATGTGATGCTGCTGACCTCTGACGCCCCCGAGTATAAGCCTTGGGCCCTGGTCATCCAGGATTCTAACGGCGAGAATAAGATCAAGATGCTGAGCGGAGGATCCGGAGGATCTGGAGGCAGCGAAGCCAGCCCAGCATCCGGGCCCAGACACTTGATGGATCCACACATATTCACTTCCAACTTTAACAATGGCATTGGAAGGCATAAGACCTACCTGTGCTACGAAGTGGAGCGCCTGGACAATGGCACCTCGGTCAAGATGGACCAGCACAGGGGCTTTCTACACAACCAGGCTAAGAATCTTCTCTGTGGCTTTTACGGCCGCCATGCGGAGCTGCGCTTCTTGGACCTGGTTCCTTCTTTGCAGTTGGACCCGGCCCAGATCTACAGGGTCACTTGGTTCATCTCCTGGAGCCCCTGCTTCTCCTGGGGCTGTGCCGGGGAAGTGCGTGCGTTCCTTCAGGAGAACACACACGTGAGACTGCGTATCTTCGCTGCCCGCATCTTCGATTACGACCCCCTATATAAGGAGGCACTGCAAATGCTGCGGGATGCTGGGGCCCAAGTCTCCATCATGACCTACGATGAATTTAAGCACTGCTGGGACACCTTTGTGGACCACCAGGGATGTCCCTTCCAGCCCTGGGATGGACTAGATGAGCACAGCCAAGCCCTGAGTGGGAGGCTGCGGGCCATTCTCCAGAATCAGGGAAACTCTGGAGGATCTAGCGGAGGATCCTCTGGCAGCGAGACACCAGGAACAAGCGAGTCAGCAACACCAGAGAGCAGTGGCGGCAGCAGCGGCGGCAGCGACAAGAAGTATAGCATCGGGCTGGCCATTGGAACGAACTCGGTTGGTTGGGCTGTGATTACGGACGAATACAAGGTGCCATCCAAGAAGTTTAAGGTCCTGGGAAACACCGACCGTCACTCAATCAAGAAGAATCTCATTGGAGCCCTGCTCTTCGATAGTGGGGAAACTGCCGAAGCTACTCGACTGA | Cloning for pGY40 |
| 52_gBlocks | ATACACGTCCACAAAGGAGGTGCTTGATGCGACCCTGATTCATCAATCCATCACTGGGCTCTATGAAACCCGTATCGACCTTAGTCAACTGGGGGGCGACAGCGCTGGAGGAGGTGGAAGCGGAGGAGGAGGAAGCGGAGGAGGAGGTAGCGGACCTAAGAAAAAGAGGAAGGTGGCGGCCGCTTAAGCGGCCGCTGAGCGGACATTCGATTTA | Cloning for pGY40 |
| 53_gBlocks | ATGGTCTCCTCCACGCTGACCAGCATGTTGACTGTTTTAGAGCTAGAAATAGCAAGTTAAAATAAGGCTAGTCCGTTATCAACTTGAAAAAGTGGCACCGAGTCGGTGCGGGGCAATGGCGCATCTGGGAGCGCGTCAGACTGAAGATCTGGAGGTGGCCGGTTCAAGCCCGGCTTGCCCCAACGACGACTATGCTGGGACAGTTTGGAGACCTA | Cloning for pGY41 |
| 74_gBlocks | GAGTATCTGAGCACTTCTCCCTTTTATATTCCACAAAACATAACACGCGGCCGCATGAAGCGGACAGCAGACGGTTCCGAGTTCGAGTCTCCTAAGAAAAAGAGGAAGGTGAGCGAGGTAGAGTTTTCGCATGAATATTGGATGCGACATGCTCTCACTCTTGCAAAACGGGCACGTGATGAAAGGGAGGTACCTGTGGGAGCGGTGCTGGTCCTCAACAATAGAGTGATCGGCGAGGGATGGAATAGAGCAATCGGCCTGCATGACCCAACAGCTCATGCTGAAATCATGGCACTCCGGCAGGGGGGCCTGGTGATGCAAAATTACCGTTTGATCGATGCGACACTCTACGTGACGTTCGAGCCGTGCGTTATGTGTGCTGGGGCCATGATTCATTCACGCATTGGAAGGGTGGTGTTTGGTTGGAGGAACTCCAAGAGGGGGGCAGCGGGCTCTTTGATGAACGTTTTGAACTATCCGGGCATGAACCATAGAGTCGAGATCACAGAGGGCATACTCGCCGACGAGTGTGCGGCTCTCCTTTGCGATTTCTACCGGATGCCAAGGCAAGTATTTAATGCACAAAAGAAGGCTCAGTCCAGTATTAACTCTGGAGGATCTAGCGGAGGATCCTCTGGCAGCGAGACACCAGGAACAAGCGAGTCAGCAACACCAGAGAGCAGTGGCGGCAGCAGCGGCGGCAGCGACAAGAAGTATAGCATCGGGCTGGCCATTGGAACGAACTCGGTTGGTTGGGCTGTGATTACGGACGAATACAAGGTGCCATCCAAGAAGTTTAAGGTCCTGGGAAACACCGACCGTCACTCAATCAAGAAGAATCTCATTGGAGCCCTGCTCTTCGATAGTGGGGAAACTGCCGAAGCTACTCGACTGA | Cloning for pGY49 |
| 75_gBlocks | ATACACGTCCACAAAGGAGGTGCTTGATGCGACCCTGATTCATCAATCCATCACTGGGCTCTATGAAACCCGTATCGACCTTAGTCAACTGGGGGGCGACTCTGGCGGAAGTAAGAGAACAGCCGATGGCTCGGAGTTTGAGAGTCCTAAGAAAAAGAGGAAGGTGGAGTAAGCGGCCGCTGAGCGGACATTCGATTTA | Cloning for pGY49 |
| 76_gBlocks | GATGGTCTCCTCCACAGACCAGCGACATCGAAGCGTTTTAGAGCTAGAAATAGCAAGTTAAAATAAGGCTAGTCCGTTATCAACTTGAAAAAGTGGCACCGAGTCGGTGCGGGGCAATGGCGCATCTGGGAGCGCGTCAGACTGAAGATCTGGAGGTGGCCGGTTCAAGCCCGGCTTGCCCCAGGGAAGAGCTTCCGATGAGAGTTTGGAGACCTA | Cloning for pGY50 |
| 88_gBlocks | ATGGTCTCCTCCAATATACGGTTTCGAAGAAGGGTTTTAGAGCTAGAAATAGCAAGTTAAAATAAGGCTAGTCCGTTATCAACTTGAAAAAGTGGCACCGAGTCGGTGCGGGGCAATGGCGCATCTGGGAGCGCGTCAGACTGAAGATCTGGAGGTGGCCGGTTCAAGCCCGGCTTGCCCCATTCGAAACCGTATATCAGCGGTTTGGAGACCTA | Cloning for pGY60 |
| 89_gBlocks | ATGGTCTCCTCCATCTGTCAGCATCCCACAATGGTTTTAGAGCTAGAAATAGCAAGTTAAAATAAGGCTAGTCCGTTATCAACTTGAAAAAGTGGCACCGAGTCGGTGCGGGGCAATGGCGCATCTGGGAGCGCGTCAGACTGAAGATCTGGAGGTGGCCGGTTCAAGCCCGGCTTGCCCCAAATTCATCAAGTACCGTAGGGTTTGGAGACCTA | Cloning for pGY61 |
| 137_gBlocks | CGGCCAAAGCGTAACTCTGACAAGCTGATCGCTCGAAAGAAGGATTGGGACCCCAAGAAGTATGGAGGGTTCGTTTCTCCTACAGTGGCATACTCGGTTCTCGTTGTCGCGAAGGTTGAGAAGGGAAAGTCTAAGAAGCTGAAGTCGGTCAAGGAACTGCTCGGGATCACCATTATGGAGCGCTCCAGCTTCGAAAAGAATCCCATCGACTTTCTCGAGGCCAAGGGCTATAAGGAAGTCAAGAAGGATCTTATCATTAAGCTGCCTAAGTACTCTTTGTTCGAGCTTGAAAACGGTCGAAAGCGAATGCTCGCATCGGCACGATTCTTGCAGAAGGGGAATGAATTGGCACTTCCCTCAAAGTACGTGAACTTCCTGTATCTCGCGTCCCACTACGAGAAGCTGAAGGGTAGCCCTGAGGACAACGAACAGAAGCAACTTTTTGTTGAGCAACACAAGCATTATCTGGATGAGATCATTGAACAGATTTCAGAGTTCAGTAAGCGCGTCATCCTCGCCGATGCTAATCTCGACAAGGTGTTGTCGGCCTACAACAAGCACCGTGACAAGCCGATCCGAGAGCAGGCTGAAAATATCATTCATCTGTTCACCCTCACTAACTTGGGAGCACCACGAGCGTTCAAGTATTTTGATACGACAATCGACCGTAAGGTGTACCGA | Cloning for pGY150 and pGY151 |

**Supplementary** **Table S2. All DNA oligos used in this study.**

| **Name** | **Sequence** | **Purpose** |
| --- | --- | --- |
| 162_F | GGGAGAAGTGCTCAGATACT | Cloning for pGY40 and pGY49 |
| 185_R | GGGGTTCGATTCCCCGCAGC | Cloning for pGY40, pGY49, pGY150 and pGY151 |
| 67_F | GCCGAAGCTACTCGACTGA | Cloning for pGY40 and pGY49 |
| 166_R | CCTCCTTTGTGGACGTGTAT | Cloning for pGY40 and pGY49 |
| 165_R | CTGAGCGGACATTCGATTT | Cloning for pGY40 and pGY49 |
| 186_F | ATGTCTTGAATCGCGCATTG | Cloning for pGY40, pGY49, pGY150 and pGY151 |
| 426_F | TCAGAGTTACGCTTTGGCCGAATCGATTCCTTAGAGAAACCG | Cloning for pGY150 and pGY151 |
| 427_F | TCGACCGTAAGGTGTACCGATCCACAAAGGAGGTGCTT | Cloning for pGY150 and pGY151 |
| 256_F | ATGGTCTCCTCCATCTGTCAGCATCCCACAATGGTTTGGAGACCTA | Cloning for pGY71 |
| 257_R | TAGGTCTCCAAACCATTGTGGGATGCTGACAGATGGAGGAGACCAT | Cloning for pGY71 |
| 312_F | ATGGTCTCCTCCAGTCAGCATCCCACAATGCGGGTTTGGAGACCTA | Cloning for pGY84 |
| 313_R | TAGGTCTCCAAACCCGCATTGTGGGATGCTGACTGGAGGAGACCAT | Cloning for pGY84 |
| 314_F | ATGGTCTCCTCCATGTCAGCATCCCACAATGCGGTTTGGAGACCTA | Cloning for pGY85 |
| 315_R | TAGGTCTCCAAACCGCATTGTGGGATGCTGACATGGAGGAGACCAT | Cloning for pGY85 |
| 316_F | ATGGTCTCCTCCAACCAGCCTTCAGCTTTTACGGTTTGGAGACCTA | Cloning for pGY86 |
| 317_R | TAGGTCTCCAAACCGTAAAAGCTGAAGGCTGGTTGGAGGAGACCAT | Cloning for pGY86 |
| 318_F | ATGGTCTCCTCCAACGCAGTCGTTGGGCAAGAGGTTTGGAGACCTA | Cloning for pGY87 |
| 319_R | TAGGTCTCCAAACCTCTTGCCCAACGACTGCGTTGGAGGAGACCAT | Cloning for pGY87 |
| 320_F | ATGGTCTCCTCCAAATCTTATGCAACTGAGCGCGTTTGGAGACCTA | Cloning for pGY88 |
| 321_R | TAGGTCTCCAAACGCGCTCAGTTGCATAAGATTTGGAGGAGACCAT | Cloning for pGY88 |
| 322_F | ATGGTCTCCTCCAGAATCTTATGCAACTGAGCGGTTTGGAGACCTA | Cloning for pGY89 |
| 323_R | TAGGTCTCCAAACCGCTCAGTTGCATAAGATTCTGGAGGAGACCAT | Cloning for pGY89 |
| 324_F | ATGGTCTCCTCCATGTGGGATGCTGACAGATGCGTTTGGAGACCTA | Cloning for pGY90 |
| 325_R | TAGGTCTCCAAACGCATCTGTCAGCATCCCACATGGAGGAGACCAT | Cloning for pGY90 |
| 326_F | ATGGTCTCCTCCACGAATCTTAACGCAGTCGTTGTTTGGAGACCTA | Cloning for pGY91 |
| 327_R | TAGGTCTCCAAACAACGACTGCGTTAAGATTCGTGGAGGAGACCAT | Cloning for pGY91 |
| 328_F | ATGGTCTCCTCCAAACTTTAAACCCCCGCATTGGTTTGGAGACCTA | Cloning for pGY92 |
| 329_R | TAGGTCTCCAAACCAATGCGGGGGTTTAAAGTTTGGAGGAGACCAT | Cloning for pGY92 |
| 332_F | ATGGTCTCCTCCAAGATTCAAGGTGCTAATCATGTTTGGAGACCTA | Cloning for pGY94 |
| 333_R | TAGGTCTCCAAACATGATTAGCACCTTGAATCTTGGAGGAGACCAT | Cloning for pGY94 |
| 334_F | ATGGTCTCCTCCATCAGACTTGTCAAGTATAGAGTTTGGAGACCTA | Cloning for pGY95 |
| 335_R | TAGGTCTCCAAACTCTATACTTGACAAGTCTGATGGAGGAGACCAT | Cloning for pGY95 |
| 336_F | ATGGTCTCCTCCACTGTCAGCATCCCACAATGCGTTTGGAGACCTA | Cloning for pGY96 |
| 337_R | TAGGTCTCCAAACGCATTGTGGGATGCTGACAGTGGAGGAGACCAT | Cloning for pGY96 |
| 338_F | ATGGTCTCCTCCAACAATGCGGGGGTTTAAAGTGTTTGGAGACCTA | Cloning for pGY97 |
| 339_R | TAGGTCTCCAAACACTTTAAACCCCCGCATTGTTGGAGGAGACCAT | Cloning for pGY97 |
| 340_F | ATGGTCTCCTCCAGCTCAGTTGCATAAGATTCAGTTTGGAGACCTA | Cloning for pGY98 |
| 341_R | TAGGTCTCCAAACTGAATCTTATGCAACTGAGCTGGAGGAGACCAT | Cloning for pGY98 |
| 342_F | ATGGTCTCCTCCAACTTTAAACCCCCGCATTGTGTTTGGAGACCTA | Cloning for pGY99 |
| 343_R | TAGGTCTCCAAACACAATGCGGGGGTTTAAAGTTGGAGGAGACCAT | Cloning for pGY99 |
| 344_F | ATGGTCTCCTCCACGTTAAGATTCGTACTAATCGTTTGGAGACCTA | Cloning for pGY100 |
| 345_R | TAGGTCTCCAAACGATTAGTACGAATCTTAACGTGGAGGAGACCAT | Cloning for pGY100 |
| 346_F | ATGGTCTCCTCCATTCAGCTTTTACGGGGATCTGTTTGGAGACCTA | Cloning for pGY101 |
| 347_R | TAGGTCTCCAAACAGATCCCCGTAAAAGCTGAATGGAGGAGACCAT | Cloning for pGY101 |
| 348_F | ATGGTCTCCTCCAGACCAGCCTTCAGCTTTTACGTTTGGAGACCTA | Cloning for pGY102 |
| 349_R | TAGGTCTCCAAACGTAAAAGCTGAAGGCTGGTCTGGAGGAGACCAT | Cloning for pGY102 |
| 350_F | ATGGTCTCCTCCATGACCAGCCTTCAGCTTTTAGTTTGGAGACCTA | Cloning for pGY103 |
| 351_R | TAGGTCTCCAAACTAAAAGCTGAAGGCTGGTCATGGAGGAGACCAT | Cloning for pGY103 |
| 430_F | ATGGTCTCCTCCACCCTCCATGTTTGCGGAAGAGTTTGGAGACCTA | Cloning for pGY159 and pGY164 |
| 431_R | TAGGTCTCCAAACTCTTCCGCAAACATGGAGGGTGGAGGAGACCAT | Cloning for pGY159 and pGY164 |
| 438_F | ATGGTCTCCTCCAGCACTGCGACTGGGAATCTGGTTTGGAGACCTA | Cloning for pGY163 |
| 439_R | TAGGTCTCCAAACCAGATTCCCAGTCGCAGTGCTGGAGGAGACCAT | Cloning for pGY163 |
| 440_F | ATGGTCTCCTCCACAAACATGGAGGGTCCATCTGTTTGGAGACCTA | Cloning for pGY165 |
| 441_R | TAGGTCTCCAAACAGATGGACCCTCCATGTTTGTGGAGGAGACCAT | Cloning for pGY165 |
| 442_F | ATGGTCTCCTCCAAACATGGAGGGTCCATCTCGGTTTGGAGACCTA | Cloning for pGY166 |
| 443_R | TAGGTCTCCAAACCGAGATGGACCCTCCATGTTTGGAGGAGACCAT | Cloning for pGY166 |
| 444_F | ATGGTCTCCTCCATGGTCAGACTTGTCAAGTATGTTTGGAGACCTA | Cloning for pGY167 |
| 445_R | TAGGTCTCCAAACATACTTGACAAGTCTGACCATGGAGGAGACCAT | Cloning for pGY167 |
